# Act1 is a negative regulator in T and B cells via direct inhibition of STAT3

**DOI:** 10.1038/s41467-018-04974-3

**Published:** 2018-07-16

**Authors:** Cun-Jin Zhang, Chenhui Wang, Meiling Jiang, Chunfang Gu, Jianxin Xiao, Xing Chen, Bradley N. Martin, Fangqiang Tang, Erin Yamamoto, Yibo Xian, Han Wang, Fengling Li, R. Balfour Sartor, Howard Smith, M. Elaine Husni, Fu-Dong Shi, Ji Gao, Julie Carman, Ashok Dongre, Susan C. McKarns, Ken Coppieters, Trine N. Jørgensen, Warren J. Leonard, Xiaoxia Li

**Affiliations:** 1 Department of Inflammation and Immunity, Lerner Research Institute, Cleveland Clinic, Cleveland, OH 44106 USA; 2 Department of Neurology, Tianjin Neurological Institute, Tianjin Medical University General Hospital, Tianjin, 300051 China; 3 Center for Neuroinflammation, Beijing Tiantan Hospital, Capital Medical University, Beijing, 100050 China; 4 Key Laboratory of Molecular Biophysics of the Ministry of Education, College of Life Science and Technology, Huazhong University of Science and Technology, Wuhan, 430074 China; 5 Wuhan Institute of Biotechnology, Wuhan, 430200 China; 6 Institute of Radiation Medicine, Chinese Academy of Medical Sciences and Peking Union Medical College, Tianjin, 300192 China; 7 National Gnotobiotic Rodent Resource Center, Department of Medicine and Center for Gastrointestinal Biology and Disease, University of North Carolina, Chapel Hill, NC 27599 USA; 8 Department of Microbiology and Immunology, University of North Carolina, Chapel Hill, NC 27599 USA; 9 Department of Rheumatologic and Immunologic Disease, Cleveland Clinic, Cleveland, OH 44106 USA; 10 Department of Neurology, Barrow Neurological Institute, St. Joseph’s Hospital and Medical Center, Phoenix, AZ 85013 USA; 11 Discovery Biology, Bristol-Myers Squibb, Princeton, NJ 08540 USA; 12 Department of Surgery, University of Missouri School of Medicine, Columbia, MO 65212 USA; 13 Department of Molecular Microbiology and Immunology, University of Missouri School of Medicine, Columbia, MO 65212 USA; 14 Type 1 Diabetes Center, Novo Nordisk A/S, Søborg, 2860 Denmark; 15 Laboratory of Molecular Immunology and the Immunology Center, National Heart, Lung, and Blood Institute, National Institutes of Health, Bethesda, MD 20892 USA

## Abstract

Although Act1 (adaptor for IL-17 receptors) is necessary for IL-17-mediated inflammatory responses, *Act1*- (but not *Il17ra-*, *Il17rc-*, or *Il17rb*-) deficient mice develop spontaneous SLE- and Sjögren’s-like diseases. Here, we show that Act1 functions as a negative regulator in T and B cells via direct inhibition of STAT3. Mass spectrometry analysis detected an Act1–STAT3 complex, deficiency of *Act1* (but not *Il17ra*-, *Il17rc-*, or *Il17rb*) results in hyper IL-23- and IL-21-induced STAT3 activation in T and B cells, respectively. IL-23R deletion or blockade of IL-21 ameliorates SLE- and Sjögren’s-like diseases in *Act1*^*−/−*^ mice. Act1 deficiency results in hyperactivated follicular Th17 cells with elevated IL-21 expression, which promotes T–B cell interaction for B cell expansion and antibody production. Moreover, anti-IL-21 ameliorates the SLE- and Sjögren’s-like diseases in Act1-deficient mice. Thus, IL-21 blocking antibody might be an effective therapy for treating SLE- and Sjögren’s-like syndrome in patients containing Act1 mutation.

## Abstract (web summary)

Adaptor for IL-17 receptors (Act1) is known to be crucial for IL-17-mediated immune responses. Here the authors show that Act1 also functions as a negative regulator of T and B cells by direct inhibition of STAT3.

## Introduction

Systemic lupus erythematosus (SLE) is a complex, chronic systemic autoimmune disorder with a heterogeneous presentation commonly targeting joints, skin, hematologic, and kidneys without a known cure^1,2^. Th17 cells are a population of proinflammatory CD4^+^ effector T cells that produce interleukin 17A (IL-17A), IL-17F, IL-21, and IL-22. While Th17 cells play important roles in immune homeostasis and host defense, an overactive Th17 response has been implicated in various inflammatory and autoimmune conditions including psoriasis, SLE, and Sjögrens syndrome^3–9^.

The signaling cascade of IL-17, the signature cytokine of Th17 cells, requires a key signaling molecule, Act1 (also known as TRAF3IP2 or CIKS) to propagate downstream signaling events in tissue cells, including activation of the transcription factor NF-κB^10–13^. The absence of Act1 leads to resistance to IL-17-mediated inflammation in mouse models of experimental autoimmune encephalomyelitis (EAE) and asthma^10,14–16^. Although Act1 is necessary for IL-17-mediated inflammatory responses, *Act1*^*−/−*^ mice develop hyper Th17 responses (with increased IL-17 producing CD4^+^ T cells in lymph nodes and spleen) and spontaneous inflammatory/autoimmune diseases, including skin inflammation, SLE-like nephritis, and Sjögren’s-like disease^3–6^. Notably, multiple genome-wide association studies have linked a variant of Act1 with substitution of asparagine for aspartic acid at position 10 (SNP-D10N) to susceptibility to psoriasis and SLE^17–20^. We reported that ACT1^D10N/D10N^ T cells exhibit a dysregulated and hyperactive Th17 response, implicating an intricate mechanism by which this single nucleotide polymorphism can be linked to human disease^3,21^. Supporting cell-specific effects, we demonstrated that the hyperactive Th17 response in Act1^−/−^ mice was T cell intrinsic. One critical question is whether the hyper Th17 response in *Act1*^*−/*−^ mice is required for the SLE-like nephritis and Sjögren’s-like disease associated with Act1 deficiency. Furthermore, the molecular mechanism of how Act1 deficiency results in hyper Th17 response remains unclear. Notably, although a hyper Th17 response (elevated IL-17) was also observed in *Il17ra*^*−/*−^ and *Il17rc*^*−/*−^ mice, SLE- and Sjögren’s-like diseases were not observed in these mice, indicating that the autoimmune phenotype in *Act1*^*−/*−^ mice is probably not simply due to lack of IL-17 signaling. In support of this, whereas the hyperactive Th17 response in *Act1*^−/−^ mice was T cell intrinsic^3^, the hyperactive Th17 response associated with *IL-17RA* deficiency was not observed in T cell-specific IL-17RA-deficient mice^22^.

In this study, we report that Act1 plays a critical role in modulating Th17 polarization via direct inhibition of STAT3. Mass spectrometry analyses followed by co-immunoprecipitation showed that Act1 (but not the SNP-D10N mutant) was able to directly interact with and suppress STAT3 activation in Th17 cells. Deficiency of *Act1* (but not *Il17ra*-, *Il17rc-*, or *Il17rb*) results in hyper IL-23-induced STAT3 activation in naive CD4^+^ T cells and increases IL-21 expression. IL-23R deletion reduces Th17 cells and ameliorates autoimmune diseases in *Act1*^*−/*−^ mice, implicating the importance of hyper Th17 cells (with increased STAT3 activation and IL-21 expression) for the autoimmune diseases associated with Act1 deficiency. Furthermore, deficiency of *Act1* (but not *Il17ra*-, *Il17rc-*, or *Il17rb*) results in hyper IL-21-induced STAT3 activation in B cells. Deletion of IL-21R or blockade of IL-21 with an anti-IL-21 neutralizing antibody ameliorates the development of Sjögren’s and SLE-like diseases in *Act1*^*−/*−^ mice. These findings indicate that Act1 modulates IL-23/IL-21-dependent autoimmunity via suppression of STAT3 activation, providing a mechanism for the association of the SNP-D10N mutation with SLE.

## Results

### Act1 suppresses IL-23-induced STAT3 activation in Th17 cells

**Fig. 1:**
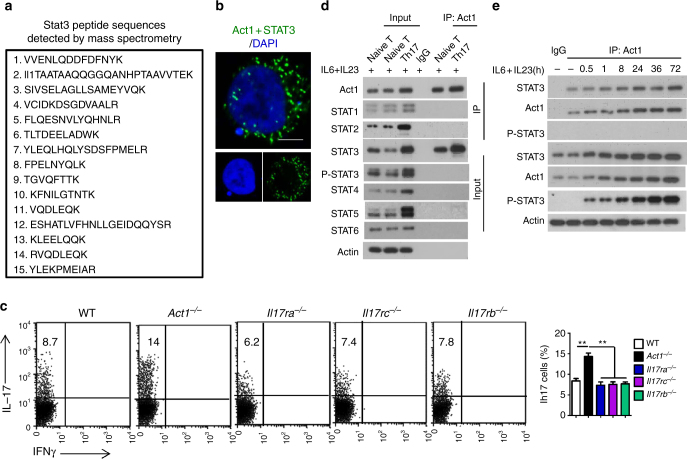
Act1 physically interacts with STAT3. **a** Mass spectrometry analysis of Act1-associated proteins after immunoprecipitation via anti-Flag beads from lysates of HeLa cells transiently transfected to express Act1-Flag. Fifteen matched peptide sequences that correspond to STAT3 were identified. **b** HeLa cells were co-transfected with V5-Act1 and Flag-STAT3, followed by Duolink assay, in which mouse anti-V5 and rabbit anti-Flag antibody were used. Green dots present the interaction of STAT3 and Act1. Scale bars: 10 μm. **c** Naive T cells isolated from spleens of indicated mice were polarized to Th17 with IL-23 + IL-6 for 3 days, followed by intracellular staining for IL-17A and IFNγ. **d** WT Naive T cells isolated from spleen were polarized into Th17 cells with IL-23 + IL-6. Lysates were then immunoprecipitated with anti-Act1 followed by western analysis of indicated proteins. **e** Naive CD4^+^ T cells were stimulated with IL-6 + 23 for the indicated time. Cells were then lysed and immunoprecipitated with anti-Act1 followed by western analysis with the indicated antibodies. Graphed as mean ± SEM. **p < 0.01. *N* = 4–6/group. Two-tailed Student’s *T* test. All the data presented were from three independent experiments

We previously reported that the hyperactive Th17 response in *Act1*^−/−^ mice was T cell intrinsic^3^, whereas the hyperactive Th17 response associated with *Il17ra* deficiency was not observed in T cell-specific *Il17ra*-deficient mice^22^. Thus, we hypothesize that Act1 may play a direct role in modulating Th17 polarization, which is independent of IL-17 signaling. Mass spec analysis of Act1-coimmunoprecipitates showed that STAT3 is a potential Act1-interacting protein (Fig. 1a and Supplementary Fig. 1a). We validated the interaction between Act1 and STAT3 by immunoprecipitation-western analysis and proximity ligation assay (Fig. 1b, d, e). IL-23 and IL-6 are the key cytokines for inducing human and murine Th17 cell differentiation/expansion by activating STAT3^23,24^. When naive T cells were polarized into Th17 cells by IL-23 and IL-6, deficiency of Act1 in T cells resulted in increased IL-17^+^CD4^+^ T cells (Fig. 1c), whereas Act1 deficiency had no impact on Th1 or Th2 cell polarization (Supplementary Fig. 1b, c). Consistently, the hyper Th17 phenotype was also observed in Act1 knockdown cells (Supplementary Fig. 1d, e). On the other hand, deficiency of *Il17ra*, *Il17rc*, or *Il17rb* had no impact on the polarization of naive CD4^+^ T cells into Th17 cells ex vivo (Fig. 1c). While Act1 expression was induced during Th17 cell polarization by IL-23/IL-6, the endogenous Act1 formed a complex with STAT3, but not with other STATs, in Th17 cells, implicating a potential role for STAT3 in Act1-mediated modulation of Th17 cells (Fig. 1d). Notably, phosphorylated STAT3 was not detected in Act1-immunoprecipitates, suggesting that Act1 probably formed a complex with unphosphorylated STAT3 (Fig. 1d, e).

**Fig. 2:**
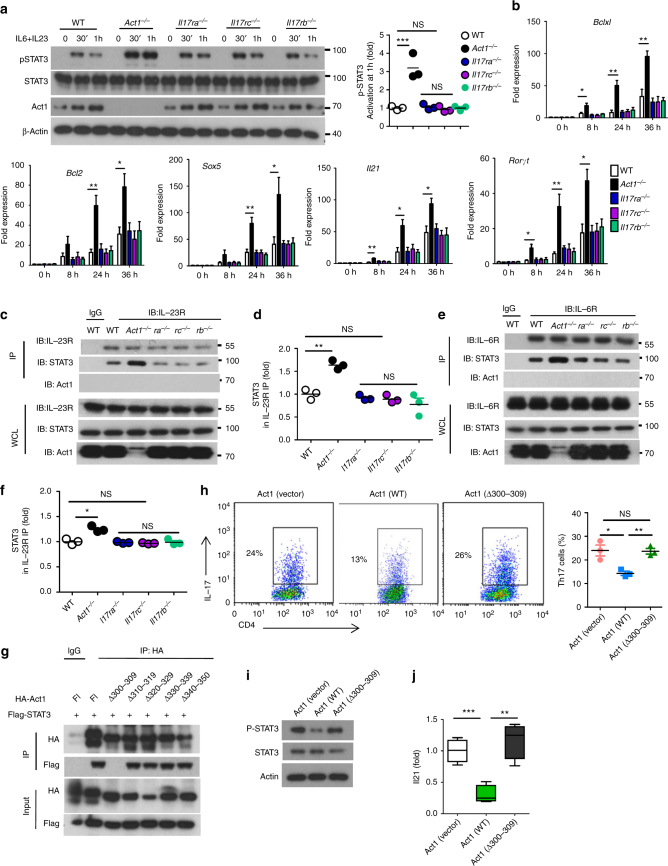
Act1 competes with IL-23R for STAT3 binding and negatively regulates STAT3 activation. **a** Naive T cells stimulated with IL-23 + IL-6 for the indicated times, followed by western analysis of indicated proteins. STAT3 phosphorylation was quantified as a ratio of phosphorylated-to-total STAT3. Data presented as fold of induction of the cells from knockouts over the wild-type cells. **b** RT-PCR analysis of STAT3-target genes in Naive T cells stimulated with IL-23 + IL-6 for the indicated times. **c**–**f** Naive T cells were stimulated with IL-23 and IL-6 for 1 h, followed by immunoprecipitation with anti-IL23R (**c**, **d**) or anti-IL6R (**e**, **f**) and western analysis of indicated proteins. STAT3 levels of the immunoprecipitates were quantified as percentage of total STAT3 in the cell lysates. **g** HeLa cells were transiently co-transfected full-length STAT3 with control vector, Flag-tagged full-length Act1 (fl), or Act1 deletion mutants. Lysates were immunoprecipitated with anti-HA (HA-Act1), followed by western analysis with the indicated antibodies. **h**–**j** WT-Act1 and Act1∆300–309 mutant in retroviral vector (carrying RPF) were introduced into the primary *Act1*^*−/−*^ naive T cells. Sorted RFP^+^ naive T cells were polarized to Th17 cells in the presence of IL-6 + IL-23 and quantified as percentage of CD4^+^ T cells (**h**), western analysis of indicated proteins (**i**), and RT-PCR analysis of IL-21 gene expression (**j**). Graphed as mean ± SEM. *p < 0.05, **p < 0.01, *** *p* < 0.001. *N* = 4–6/group. Two-tailed Student’s *T* test. All the data presented were from three independent experiments

We then examined IL-23 and IL-6 signaling in wild-type and *Act1*-deficient naive CD4^+^ T cells. Act1 deficiency enhanced IL-23/IL-6-induced STAT3 phosphorylation (but not p-STAT4 or p-STAT5) in naive CD4^+^ T cells (Fig. 2a and Supplementary Fig. 1f, g). Consistent with the fact that Act1 deficiency had no impact on IL-4-polarized Th2 cells, IL-4-induced phosphorylation of STAT5 and STAT6 were not affected by Act1 deficiency (Supplementary Fig. 1h). In agreement with the hyper STAT3 activation, Act1 deficiency indeed enhanced IL-23/IL-6-induced expression of STAT3-target genes, including *Bclxl*, *Bcl2*, *Sox5*, *Il21*, and* Rorγt* (Fig. 2b and Supplementary Fig. 1i). On the other hand, *Il17ra*, *Il17rc*, or *Il17rb* deficiency had no impact on IL-23/IL-6-induced STAT3 phosphorylation or the expression of STAT3-target genes in naive CD4^+^ T cells (Fig. 2a, b). Importantly, the IL-6R and IL-23R levels were comparable between wild-type and *Act1*-deficient Th17 cells. Instead, the interaction of STAT3 to IL-23R, and to a lesser degree to IL-6R, was increased in *Act1*-deficient (but not *Il17ra*, *Il17rc*, or *Il17rb*-deficient) naive CD4^+^ T cells compared to the wild-type cells in response to IL-23 or IL-6 stimulation (Fig. 2c–f and Supplementary Fig. 1j). These results suggest that the Act1–STAT3 interaction may suppress IL-23/IL-6-induced STAT3 activation by attenuating STAT3's recruitment to their receptors. We found that amino acid residues 300–309 of Act1 are required for its interaction with STAT3, but not for the interaction of Act1 with other signaling molecules such as TRAFs (Fig. 2g and Supplementary Fig. 2a). Furthermore, wild-type Act1, but not Act1 mutant (∆300–309), inhibited the interaction of STAT3 with IL-23R and IL-6R (Supplementary Fig. 2b–e). We have introduced WT-Act1 and Act1∆300–309 mutant in retroviral vector into the primary *Act1*^−/−^ naive T cells, followed by polarization to Th17 in the presence of IL-6+IL-23. The results showed that the re-introduction of WT-Act1 but not Act1∆300–309 mutant corrected the hyper T phenotype of *Act1*-deficient cells (Fig. 2h–j). Taken together, our results suggest that Act1 suppresses IL-23/IL-6-induced STAT3 activation thereby modulating Th17 polarization. Since deficiency of *Il17ra*, *Il17rc*, or *Il17rb* had no impact on STAT3 activation or the polarization of naive CD4^+^ T cells into Th17 cells ex vivo, our results indicate that the modulation of Th17 cells by the Act1–STAT3 axis is independent of IL-17 signaling.

### IL-23R is required for the autoimmunity in *Act1*^−/−^ mice

**Fig. 3:**
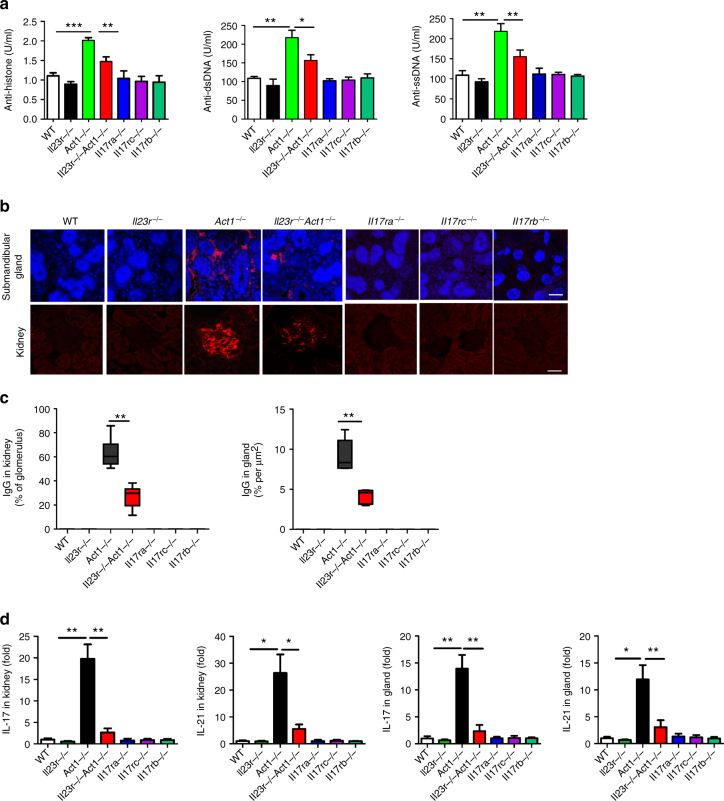
IL-23R is required for the autoimmune diseases in *Act1*^−/−^ mice. **a** Serum samples were obtained from 8-month-old mice with indicated phenotypes and analyzed for levels of anti-histone, anti-dsDNA, and anti-ssDNA lgG. **b** Kidneys and submandibular glands were harvested and quick frozen in OCT, followed by immunofluorescence staining for IgG (red). Scale bars, 50 µm. **c** Quantification of lgG deposit in glomerulus of kidney and submandibular gland. **d** RT-PCR analysis of IL-17 and IL-21 transcripts in submandibular gland and kidney from 8-month-old mice with indicated genotypes. *N* = 4–6/group. Mean ± SEM. *p < 0.05; **p < 0.01; ***p < 0.001; Two-tailed Student’s *T* test. All the data presented were from two independent experiments

Although Act1 is necessary for IL-17-mediated inflammatory responses, *Act1*-deficient mice develop spontaneous inflammatory/autoimmune diseases, including hypergammaglobulinemia, elevated serum autoantibodies, SLE-like nephritis, and Sjögren’s-like disease^3–6^. Since *Act1*-deficient T cells showed hyper IL-23R signaling, we generated *Act1*^−/−^ mice also deficient in the receptor for IL-23 (*Act1*^−/−^*Il23r*^−/−^ mice). The *Act1*^−/−^*Il23r*^−/−^ showed reduced Th17 cells in the spleen of *Act1*^−/−^*Il23r*^−/−^ mice as compared with *Act1*^−/−^ mice (Supplementary Fig. 3a–d). Notably, Act1 expression was not altered in native CD4 T cells and B cells from wild-type and *Il23r*^−/−^ mice (Supplementary Fig. 3a). Furthermore, *Act1*^−/−^*Il23r*^−/−^ mice showed reduced anti-histone, anti-dsDNA and anti-ssDNA IgG production compared to that of littermate control *Act1*^*−/−*^ mice, albeit not to the WT level (Fig. 3a). Kidneys and submandibular glands were harvested from 8-month-old wild-type, *Act1*^−/−^, *Il23r*^−/−^, and *Act1*^−/−^*Il23r*^−/−^ mice, and analyzed for IgG-immune complex deposits by immunofluorescence staining. Reduced IgG deposits and inflammation were observed in *Act1*^*−/−*^*Il23r*^*−/−*^ as compared with *Act1*^*−/−*^ mice (Fig. 3b,c and Supplementary Fig. 3e). Th17 signature mRNAs, including IL-17 and IL-21, were also significantly downregulated in the kidneys and glands of *Act1*^*−/−*^*Il23r*^*−/−*^ mice (Fig. 3d). Consistent with reduced inflammation, flow cytometry showed that *Act1*^*−/−*^*Il23r*^*−/−*^ kidneys and submandibular glands had much diminished levels of CD4^+^ T cells and B220^+ ^B cells compared to that of littermate control *Act1*^*−/−*^ mice (Supplementary Fig. 3f–h). Taken together, these results indicate that IL-23R is required for the SLE- and Sjögren’s-like diseases associated with Act1 deficiency. Importantly, Sjögren’s-like and SLE-like diseases were not observed in the *Il17ra*^*−/−*^, *Il17rc*^*−/−*^, and *Il17rb*^*−/−*^ mice (Fig. 3a–d). These results indicate that the autoimmune phenotype in *Act1*^−/−^ mice is probably independent of the role of Act1 in IL-17 signaling.

### IL-21R is required for the autoimmunity in *Act1*^−/−^ mice

**Fig. 4:**
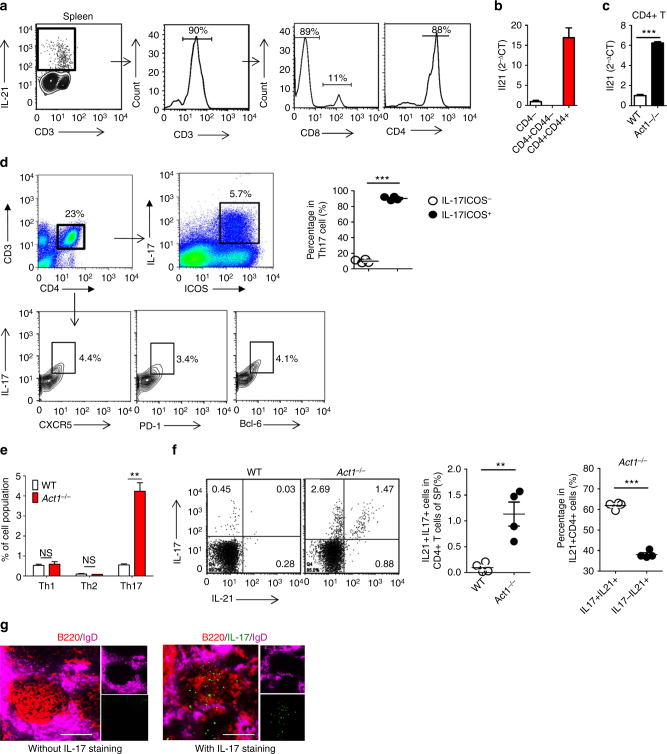
Th17 cells are the major sources of IL-21 in *Act1*^−*/*−^ mice. **a** Flow cytometry analysis of IL-21^+^CD3^+^, IL-21^+^CD4^+^, and IL-21^+^CD8^+^ T cells in spleen from 8 months old *Act1*^*−/*−^ mice. **b**,**c** RT-PCR analysis of IL-21 transcript in the sorted CD4^−^, CD4^+^CD44^−^ and CD4^+^CD44^+^ T cells from spleen of 8 months old *Act1*^−*/*−^ mice (**b**) and CD4^+^ T cells from spleen of WT or *Act*^−*/*−^ mice (**c**). **d** Flow cytometry analysis of ICOS^+^IL17^+^ Th17 cells and other Tfh markers (CXCR5, PD-1, and Bcl-6) in spleen of 8 months old *Act*^*−/−*^ mice. **e** Flow cytometry analysis of Th1 (CD3^+^CD4^+^IFNγ ^+^), Th2 (CD3^+^CD4^+^IL-4^+^), and Th17 (CD3^+^CD4^+^IL17A^+^) cells in spleen of 8 months old *Act1*^*−/−*^ mice and WT control mice. **f** Flow cytometry analysis of CD3^+^CD4^+^IL-17^+^IL-21^+^ cell in spleen of 8 months old mice. **g** Spleens collected from Act1^−/−^ mice were stained with antibodies against B220, IL-17, and lgD. Scale bars: 100 μm. *N* = 4–7/group. Mean ± SEM. **p < 0.01, ***p < 0.001. Two-tailed Student’s *T* test. All the data presented were from two independent experiments

We further investigated the molecular and cellular mechanism for how IL-23R-dependent Th17 response promotes autoimmunity in *Act1*-deficient mice. Notably, along with IL-17 and IL-22, IL-21 production was highly enhanced in Th17 cells from the spleen of *Act1*^*−/−*^ mice (Supplementary Fig. 3d). We found that CD4^+^ T cells, but not CD4^−^ cells, were the main source of IL-21 production in *Act1*^*−/−*^ mice (Fig. 4a–c). While Tfh cells are considered as a major source of IL-21^25–27^, Th17 cells can acquire features of Tfh cells^28–31^. We indeed found majority of Th17 cells express ICOS, a key marker of Tfh cells^32–36^ (Fig. 4d and Supplementary Fig. 3i). Additional Tfh cell markers (including CXCR5, PD-1, and Bcl6) were also highly expressed in CD4^+^IL-17^+^ cells from Act1^−/−^ mice (Fig. 3d and Supplementary Fig. 3i). Consistently, flow analysis indicated that Th17 with the features of Tfh cells (also called Tfh17) are the major source of IL-21 production in *Act1*^*−/*−^ spleen (Fig. 4e, f and Supplementary Fig. 4a). Moreover, IL-17^+^ cells were detected inside the B cell follicles in *Act1*-deficient spleen, demonstrating the presence of IL-17^+^ Tfh cells (referred as Tfh17 cells) (Fig. 4g).

**Fig. 5:**
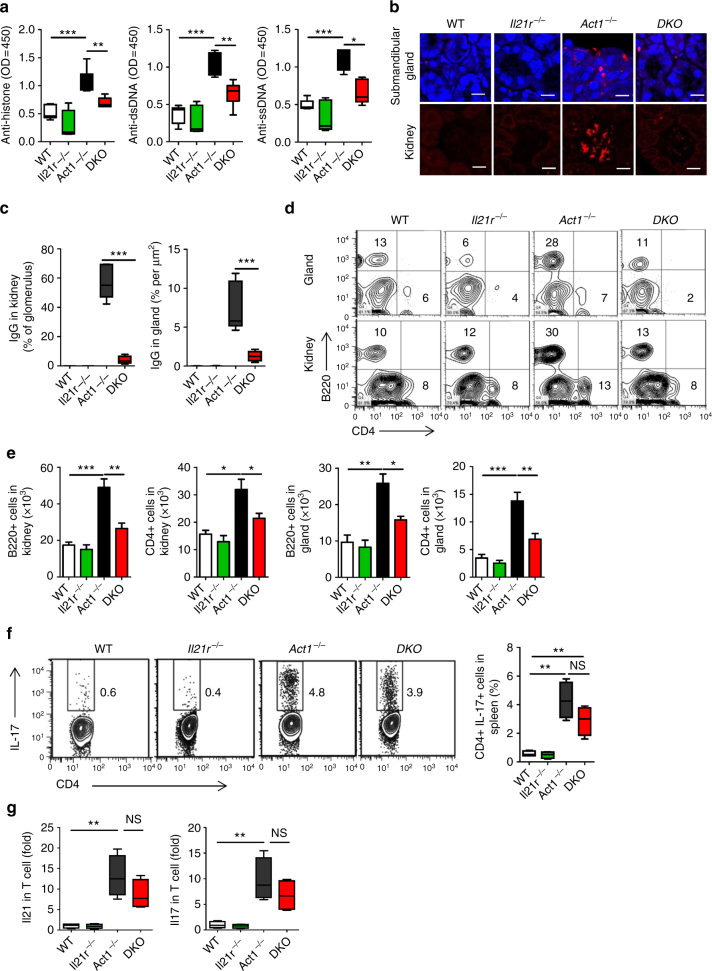
IL-21R required for the autoimmune diseases in *Act1*^−/−^ mice. All the experiments were performed on C57B/L6J background 7-month-old wild-type, *Act1*^*−/−*^, *Il21r*^*−/−*^, and *Act1*^*−/−*^ *Il21r*^*−/−*^ mice housed in SPF facility. **a** Serum samples were obtained and analyzed for levels of anti-histone, anti-dsDNA, and anti-ssDNA lgG. **b** Kidneys and submandibular glands were harvested and quick frozen in OCT, followed by immunofluorescence staining for IgG (red). Scale bars, 50 µm. **c** Quantification of lgG deposit in glomerulus of kidney and submandibular gland. **d**, **e** Flow cytometry analysis of CD45^+^B220^+^ and CD45^+^CD4^+^ cells from kidney and submandibular gland (**d**) and quantification of CD45^+^B220^+^ cells and CD45^+^CD4^+^ cells from submandibular gland and kidney (**e**). **f** Flow cytometry analysis of CD3^+^ CD4^+^ IL-17A^+^ T cells from spleen. **g** RT-PCR analysis of IL-17 and IL-21 transcripts in CD4 T cells isolated from 8-month-old mice with indicated genotypes. *N* = 4–7/group. Mean ± SEM. *p < 0.05, **p < 0.01, ***p < 0.001. Two-tailed Student’s *T* test. All the data presented were from two independent experiments

To test the impact of IL-21 on the autoimmune phenotype in Act1-deficient mice, we generated *Act1*^−/−^*Il21r*^−/−^ mice. *Act1*^*−/−*^*Il21r*^*−/−*^ mice showed substantially reduced production of autoantibodies, including anti-histone, anti-dsDNA, and anti-ssDNA IgG compared to that of *Act1*^*−/−*^ mice (Fig. 5a). Kidneys and submandibular glands were harvested from 8-month-old wild-type, *Act1*^−/−^, *Il21r*^−/−^, and *Act1*^−/−^*Il21r*^−/−^ mice, followed by immunofluorescence staining for IgG and H&E staining. Reduced IgG deposits and inflammation were observed in *Act1*^−/−^*Il21r*^−/−^ than in *Act1*^*−/−*^ mice (Fig. 5b, c and Supplementary Fig. 4b). Flow analyses showed that *Act1*^−/−^*Il21r*^−/−^ kidneys and submandibular glands had much diminished levels of infiltrating CD4^+^ T cells and B220^+^ B cells compared to that of littermate control *Act1*^−*/−*^ mice (Fig. 5d, e). Collectively, IL-21R deficiency abolished the autoimmune SLE-like nephritis and Sjögren’s syndrome diseases associated with Act1 deficiency. Although Th17 cells showed hyper STAT3 activation in response to IL-21 stimulation (Supplementary Fig. 4d), *Act1*^−/−^*Il21r*^−/−^ showed similar levels of Th17 cells and IL-21 production in the spleen compared to that in *Act1*^−/−^ mice (Fig. 5f, g). Taken together, these data suggest that the impact of IL-21R deficiency on Act1-deficient mice is likely not through the ablation of Th17 cells.

### Th17-derived IL-21 promotes T–B cell interaction in *Act1*^−/−^ mice

**Fig. 6:**
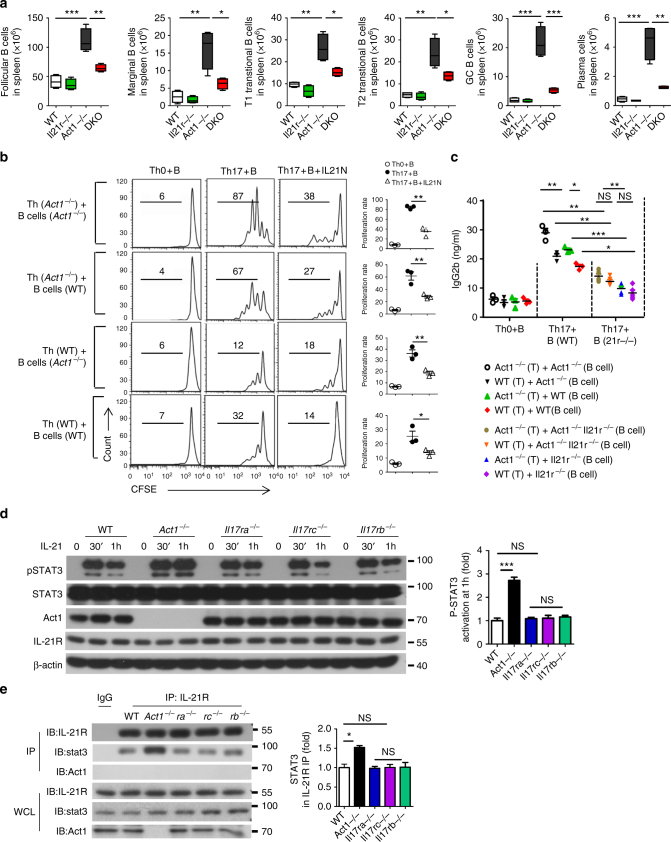
IL-21 signaling promotes B cell expansion, antibody production, and T–B cell interaction in *Act1*^*−/−*^ mice. **a** Flow cytometry analysis of marginal zone B cells (B220^+^CD21^hi^CD23^lo^), follicular B cells (B220^+^CD21^int^CD23^hi^), T1 transitional B cells (B220^+^IgM^hi^CD21^low^), T2 transitional B cells (B220^+^IgM^hi^CD21^+^), Germinal center B cell (B220^+^GL7^+^Fas^+^), and plasma cell (B220^-^CD138^+^) from spleen of C57B/L6J background 8-month-old wild-type, *Act1*^*−/−*^, *Il21r*^*−/−*^, and *Act1*^*−/−*^ *Il21r*^*−/−*^ mice. The graphs indicate the absolute cell numbers in spleen. **b** Naive T cells isolated from 6-week-old MOG_35–55_ immunized wild-type and *Act1*^*−/−*^ mice with indicated phenotypes were cultured with sorted dendritic cells from spleen of same mice in the presence of MOG_35–55_ (2 μg/ml) under Th0 or Th17-poralizing conditions, followed by co-culturing with CFSE-labeled B220^+^ B cells isolated from MOG_35–55_ immunized WT or *Act1*^*−/−*^ mice in the presence of MOG_35–55_ (2 μg/ml) with or without addition of anti-IL-21 neutralizing antibody. B cell (CD45^+^B220^+^) proliferation was assessed by CFSE dilution on day 3. **c** Naive T cells isolated from the indicated 6-week-old MOG_35–55_ immunized wild-type and *Act1*^*−/−*^ mice were cultured with sorted dendritic cells from spleen of same mice in the presence of MOG_35–55_ (2 μg/ml) under Th0 or Th17-poralizing conditions, followed by co-culturing with B cells from immunized mice with indicated genotypes in the presence of MOG_35–55_ (2 μg/ml) for 10 days followed by ELISA to determine the production of immunoglobulin in the supernatant. **d** B220^+^ B cells from WT, *Act1*^*−/−*^, *Il17ra*^*−/−*^, *Il17rc*^*−/−*^, and *Il17rb*^*−/−*^ mice were stimulated with IL-21 for the indicated times, followed by western blot analysis with the indicated antibodies. STAT3 phosphorylation was quantified as a ratio of phosphorylated-to-total STAT3. Data presented as fold of induction of the cells from knockouts over the wild-type cells. **e** B220^+^ B cells were stimulated with IL-21 for 1 h and immunoprecipitated with anti-IL21R followed by western analysis with anti-STAT3, anti-Act1, and anti-IL21R. STAT3 levels of the immunoprecipitates were quantified as percentage of total STAT3 in the cell lysates. Data presented as fold of induction of the cells from knockouts over the wild-type cells. *N* = 3–5/group. Mean ± SEM. *p < 0.05; **p < 0.01. Two-tailed Student’s *T* test. All the data presented were from two independent experiments

A prior study showed that Th17 cells can serve as effective helper cells, functioning as B-cell helpers in the induction of a proliferative response of B cells in vitro and antibody production with class switch recombination in vivo^28^. Since IL-21 is known to help B cells, we hypothesize that the elevated IL-21 from hyper Th17 cells of *Act1*^*−/−*^ mice leads to hyper B cell activation contributing to the development of Sjögren’s-like and SLE-like diseases. In support of this, we found that the expanded B cell populations observed in *Act1*^−/−^ spleens were much reduced in *Act1*^−/−^*Il21r*^−/−^ mice (Fig. 6a and Supplementary Fig. 4e, f) and *Act1*^−/−^*Il23r*^−/−^ mice (Supplementary Fig. 4c), including marginal zone B cells (B220^+^CD21^hi^CD23^lo^), follicular B cells (B220^+^CD21^int^CD23^hi^), T1 transitional B cells (B220^+^IgM^hi^CD21^low^), T2 transitional B cells (B220^+^IgM^hi^CD21^+^), germinal center B cell (B220^+^GL7^+^Fas^+^) and plasma cell (B220^−^CD138^+^). To further test this hypothesis, we examined the impact of IL-21 from Th17 cells on B cell proliferation. B220^+^ B cells and naive CD4^+^ T cells were purified from spleens of wild-type and *Act1*^*−/−*^ mice. Naive CD4^+^ T cells from wild-type or *Act1*^*−/−*^ mice were polarized to Th17 cells in vitro and added to CFSE-labeled wild-type or *Act1*^*−/−*^ B220^+^ B cells isolated from spleen of MOG immunized mice in the presence or absence of a blocking anti-IL-21 antibody for 3 days. B cell proliferation was detected by flow cytometry. We found that compared to wild-type Th17 cells, *Act1*^*−/−*^ Th17 cells induced more proliferation of both wild-type and *Act1*^*−/−*^ B cells and that this response was greatly attenuated in the presence of anti-IL-21 antibody (Fig. 6b). We then examined the impact of IL-21 from Th17 cells on antibody production. Wild-type, *Act1*^*−/−*^ or *Act1*^*−/−*^*IL-21r*^*−/−*^ B cells isolated from spleen after MOG immunization were co-cultured with wild-type or *Act1*^*−/−*^ Th17 cells in the presence of MOG_35–55_-peptide, followed by the measurement for antibody production. Again, we found that *Act1*^*−/−*^ Th17 cells showed a stronger impact on antibody production compared to wild-type Th17 cells, and that this effect was significantly blocked by IL-21R deficiency in B cells (Fig. 6c).

Notably, Act1 deficiency in B cells also showed an impact on B cell proliferation and antibody production (Fig. 6b, c). While we have previously reported the regulatory role of Act1 on CD40/BAFFR signaling, we now wondered whether the Act1's ability to interact with STAT3 has any impact on IL-21-induced STAT3 activation in B cells. We indeed found that Act1 interacted with STAT3 (but not other STATs) in B220^+^ cells (Supplementary Fig. 4g). Interestingly, Act1-deficient B220^+^ B cells showed increased IL-21-induced STAT3 phosphorylation (Fig. 6d). Importantly, the IL-21R levels were comparable between wild-type and Act1-deficient B cells (Fig. 6d). On the other hand, the interaction of STAT3 to IL-21R was increased in *Act1*-deficient (but not *Il17ra*-, *Il17rc-*, or *Il17rb*-deficient) B220 B cells compared to the wild-type cells in response to IL-21 stimulation (Fig. 6e). Consistently, IL-21-enhanced B cell proliferation was increased in Act1-deficient B cells compared to that of wild-type B cells (Supplementary Fig. 4h). Furthermore, wild-type Act1, but not Act1 mutant (Δ300–309, lost STAT3 interaction), corrected the hyper B cell activation from Act1-deficient spleen in response to IL-21 (Supplementary Fig. 4i). Thus, in addition to the reported regulatory role of Act1 on CD40/BAFFR signaling, Act1 can further suppress B cell function by attenuating IL-21-induced STAT3 activation. Taken together, these results suggest that Th17-derived IL-21 helps to promote T–B cell interaction for B cell expansion and antibody production in *Act1*-deficient mice (Supplementary Fig. 5). In support of this, we found immunization with T cell-dependent antigen NP-CGG induced more germinal center formation and plasma cell expansion in Act1-deficient mice compared to that of wild-type mice. The impact of Act1 deficiency on T cell-dependent immune response was attenuated in the *Act1*^*−/−*^*IL-21r*^*−/−*^ mice (Supplementary Fig. 4j, k).

### Anti-IL-21 ameliorates the autoimmunity in *Act1*^−/−^ mice

**Fig. 7:**
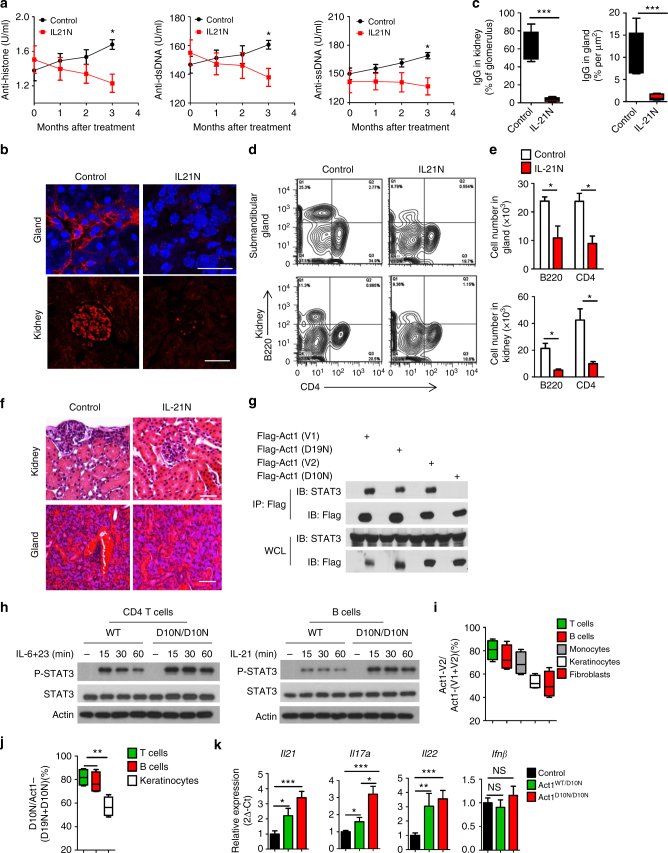
Neutralization of IL-21 attenuates the autoimmune diseases in *Act1*^*−/−*^ mice. 4-month-old SPF Balb/c *Act1*^*−/−*^ mice were treated with anti-IL21 or lgG control for additional 3 months. **a** Serum samples were obtained from *Act1*^*−/−*^ mice treated with anti-IL-21 or IgG control and analyzed for levels of anti-histone, anti-dsDNA, anti-ssDNA autoantibodies, and immunoglobulin antibodies. **b** Kidneys and submandibular glands were harvested and quick frozen in OCT, followed by immunofluorescence staining for IgG (red). Scale bars, 50 µm. **c** Quantification of lgG deposit in kidney and gland. **d** Flow cytometry analysis of CD45^+^B220^+^ cells and CD45^+^CD4^+^ cells from kidney and submandibular gland of SPF *Act1*^*−/−*^ mice treated with anti-IL-21 or IgG control. **e** Quantification of CD45^+^B220^+^ cells and CD45^+^CD4^+^ cells from submandibular gland and kidney. **f** Frozen section of kidney and gland from SPF mice were stained with H&E. **g** The indicated Act1 variants were transfected into HeLa cells, followed by co-immunoprecipitation and western analyses with the indicated antibodies. **h** Western blot analysis of IL-6 + IL-23-induced p-STAT3 in CD4^+^ T cells and IL-21-induced p-STAT3 in B cells from ACT1^WT/WT^ or ACT1^D10N/D10N^. **i**, **j** RT-PCR analysis of ACT1-v1 and ACT1-v2 in indicated human cells from control donors (**i**) or T, B cells and fibroblasts from an ACT1^*D10N/D10N*^ patient (**j**). **k** RT-PCR analysis of *Il17a*, *Il22*, *Il21*, and *Ifnβ* expression in human PBMCs from controls, ACT1^*WT/D10N*^ or ACT1^*D10N/D10N*^ individuals ACT1^*D10N/D10N*^ patients. *N* = 4–6/group. *p < 0.05, **p < 0.01, ***p < 0.001. Two-tailed Student’s *T* test. All the data presented were from two independent experiments

We then wondered whether anti-IL-21 neutralizing antibody would be effective in alleviating established disease in *Act1*^*−/−*^ mice. It is important to note that we previously reported Act1-deficient mice in Balb/c mice developed SLE- and Sjogren-like disease at the age of 3–4 months^5^. On the other hand, while SLE-like disease was also developed in Act1-deficient C57BL/6J mice around 3–4 months old, Sjogren-like disease phenotypes were only detected in Act1-deficient C57BL/6J mice at much older age (around 7–8 months old). Therefore, for both ex vivo and in vivo experiments, 7–8 months old C57BL/6J mice were used for this study. We decided to examine the therapeutic impact of anti-IL-21 on the SLE-like and Sjögren’s-like diseases in Act1-deficient Balb/c mice. Anti-IL-21 neutralizing antibody was intraperitoneally injected into 4 months old SPF *Act1*^*−/−*^ Balb/c mice every other day for 3 months. Serum autoantibody levels were substantially decreased in *Act1*^*−/−*^ mice treated with the anti-IL-21 neutralizing antibody as compared to the isotype control treated mice (Fig. 7a). Antibody deposition and cell infiltration in kidneys and submandibular glands were similarly reduced (Fig. 7b–f), showing that an IL-21 blocking antibody can be used to treat SLE-like disease and Sjögren’s-like syndrome in *Act1*^*−/−*^ mice. These results also indicate that the impact of IL-21 deficiency on the autoimmune phenotype of *Act1*^−/−^ C57BL/6J mice was confirmed by using anti-IL-21 blockage on *Act1*^−/−^ Balb/c mice. The fact that blockade of IL-21 diminished the B cell compartment and ameliorated the SLE- and Sjögren’s-like diseases in Act1-deficient Balb/c mice indicates that the Th17-IL-21 axis is operative in both strains of mice. The delayed onset of Sjogren-like disease phenotypes in Act1-deficient C57BL/6J mice compared to that of Act1-deficient Balb/c mice could be due to the strain-specific differential immune responses^37^. Future studies are required to further understand the underlining mechanism for this differential Th17 response in these two strains.

We recently investigated the effect of SNP-*D10N* mutation on the function of Act1 and how SNP-*D10N* might predispose patients to psoriasis^3,21^. Human ACT1 undergoes alternative splicing so that SNP-D10N results in an amino acid substitution at two different positions of two ACT1 isoforms (variant 1 and variant 2) to generate ACT1-v2-D10N and ACT1-v1-D19N, the latter of which contains nine additional amino acids at the N terminus. While ACT1-v2-D10N is non-functional (unable to interact with any of the signaling molecules in the IL-17 pathway), ACT1-v1-D19N retains the ability to interact with signaling molecules and is fully functional^3,21^. ACT1-v1, ACT1-v2 and ACT1-v1-D19N were able to interact with STAT3, whereas ACT1-v2-D10N failed to interact with STAT3 (Fig. 7g). In support of this, we found that hyper STAT3 phosphorylation was observed in ACT1^*D10N/D10N*^ T and B cells in response to IL-6 + IL-23 and IL-21, respectively (Fig. 7h). Importantly, although ACT1-v2-D10N and ACT1-v1-D19N are equally expressed in ACT1^*D10N/D10N*^ fibroblasts, ACT1^*D10N/D10N*^ T cells express predominantly ACT1-v2-D10N (Fig. 7i, j). As a consequence, ACT1^*D10N/D10N*^ T cells behave like Act1-deficient T cells and exhibit a dysregulated and hyperactive Th17 response. As expected ACT1^*D10N/D10N*^ and to a lesser extent ACT1^*WT/D10N*^ T cells have elevated *Il17a*, *Il22*, and *Il21* expression as compared to that in control T cells (Fig. 7k). Thus, IL-21 blocking antibody might be an effective therapy for treating SLE-like disease and Sjögren’s-like syndrome in patients containing the SNP-*D10N* mutation.

## Discussion

In this study, we report a novel function of Act1 as a negative regulator in T and B cells via direct inhibition of STAT3, thereby contributing to the development of autoimmune diseases associated with Act1 deficiency. While mass spectrometry analyses identified STAT3 as an Act1-interacting protein, deficiency of Act1 (but not IL-17RA, IL-17RC, or IL-17RB) resulted in hyper IL-23- and IL-21-induced STAT3 activation in T and B cells, respectively. Importantly, deletion of IL-23R signaling (*Il23r*^*−/−*^*Act1*^*−/−*^) ameliorated hyper Th17 response (including *Il21* expression) and the Sjögren’s and SLE-like diseases in the *Act1*^*−/−*^ mice. Although deletion of IL-21R signaling had little impact on the elevated IL-17/IL-21-producing CD4+ T cells (hyper Th17 response) in *Act1*^*−/−*^ mice, blockade of IL-21 diminished the B cell compartment and ameliorated the SLE- and Sjögren’s-like diseases in Act1-deficient mice. These findings indicate that Act1 is a critical checkpoint in immune homeostasis via negative regulation on STAT3 activation during IL-23-dependent Th17 response and IL-21-driven B cell function, controlling autoimmunity.

Notably, hyper Th17 responses (increased IL-17 producing CD4^+^ T cells) were also observed in IL-17 receptor knockout mice^3,22^, implicating a possible reciprocal relationship between IL-17 signaling and hyper Th17 responses. However, although *Il17rc*^*−/−*^ and *Il17ra*^*−/−*^ mice developed hyper Th17 cells with elevated *Il22* expression and skin inflammation, they did not develop SLE-nephritis and Sjögren’s-like disease. Importantly, T cell-specific Act1-deficient mice developed hyper Th17 responses with elevated *Il17* and *Il21* expression, whereas T cell-specific IL-17RC deficiency had no impact on Th17 cells^22^. Here we showed that Act1 interacts with STAT3, which suppresses IL-23-induced STAT3-dependent IL-21 induction and as well as IL-21-mediated STAT3 activation in B cells. Therefore, Act1-mediated negative regulation of T and B cell function is probably independent of Act1's role in IL-17 signaling. Since many autoimmune patients are and will be on anti-IL-17 therapy, it is important to segregate the impact of Act1 deficiency versus IL-17 inhibition. Based on our mechanistic studies here, we would not anticipate the SLE-like and Sjögren’s-like diseases (associated with Act1 deficiency) to be induced in patients under anti-IL-17 therapy.

While Tfh cells are considered as a major source of IL-21^25,27,34^, our results suggest that Th17 cells are the major source for increased IL-21 production in Act1-deficient mice. Notably, Th17 cells can acquire features of Tfh cells^28–31^. We indeed found majority of Act1-deficient Th17 cells express markers of Tfh cells including ICOS, PD-1 and Bcl6^32–36^. Furthermore, IL-17^+^ cells were detected inside the B cell follicles in *Act1*-deficient spleen, demonstrating the presence of IL-17^+^ Tfh cells (referred as Tfh17 cells). Tfh17 cells can serve as effective helper cells, functioning as B-cell helpers in the induction of a proliferative response of B cells and antibody production^28–30^. Moreover, while we previously reported the regulatory role of Act1 on CD40/BAFFR signaling^4^, we noted here that IL-21-induced STAT3 activation was enhanced in Act1-deficient B cells. Based on these findings, we propose that while Act1 is a necessary signaling molecule for IL-17 signaling, Act1 serves as a negative regulator to modulate Th17-B cell interaction via its impact on IL-23/IL-21/STAT3 axis and CD40, thereby controlling autoimmunity (Supplementary Fig. 5). It is possible that Act1 may inhibit STAT3 activation in response to additional STAT3-activating cytokines. Future studies are required to investigate the spectrum of Act1's inhibitory role on STAT3 activation.

Genome-wide association studies have identified risk variants in the *Il21* gene for SLE, psoriasis, and psoriatic arthritis^38,39^. IL-21 has been shown to have a pathogenic role in several lupus-prone mouse models and its blockade with anti-IL-21 reduced disease progression^40–42^. Furthermore, IL-21 serum levels were shown to positively correlate with disease severity in lupus patients^39^. The fact that either deletion of IL-21R or anti-IL-21 antibody treatment ameliorated the development of Sjögren’s and SLE-like diseases in Act1-deficient mice, providing a novel mechanism for the association of IL-21 with these autoimmune phenotypes. Importantly, TRAF3IP2 (coding for Act1) polymorphisms [rs33980500 (Act1-D10N) and rs13193677] were reported to have significant association with SLE susceptibility (*p* = 0.021, odds ratio (OR) = 1.71, and *p* = 0.046, OR = 1.73, respectively). While rs13193677 was reported in 15% lupus patients, 21.3% SLE patients contain rs33980500 (Act1-D10N), indicating a strong association between Act1 mutation (loss of function) and human lupus disease^17^. Moreover, Act1 expression was shown to be reduced in B cells from patients with Sjögren’s disease^43^. Therefore, the Act1-deficient mice represent a mouse model for lupus and SLE patients carrying Act1 mutations. Notably, although anti-IL-21 also attenuated disease in other lupus-prone mouse models, Act1 levels were not reduced in those lupus mice, indicating that IL-21 is probably modulated by multiple pathways. Moreover, hyper Th17 response (with increased IL-17/IL-21 producing CD4^+^ T cells) is not observed in all of the lupus-prone mice. Considering the heterogeneity of lupus patients, the efficacy of anti-IL-21 antibody on Act1-deficient mice implicates that this mouse strain can be further exploited as a specific model for testing anti-IL-21 as a potential therapy for SLE patients with the SNP-*D10N* Act1 mutation.

## Methods

### Mice

*Il23r*^*−/−*^ *and Act1*^*−/−*^ mice were generated in our lab^3,4^. *Il21r*^*−/−*^ mouse was a gift from Dr. Warren J. Leonard, Molecular Immunology Department of NIH. *Act1*^*−/−*^*Il23r*^*−/−*^ and *Act1*^*−/−*^*Il21r*^*−/−*^ mice on C57BL/6 background were generated and maintained at Cleveland Clinic. All the mice used in this study were C57BL/6 background females housed in SPF facility and sacrificed for analysis at the age of 8-month-old unless we indicate otherwise. All animal experiments conducted were approved by Cleveland Clinic Institutional Animal Care and Use Committee.

### Human sample collection

The human PBMC were collected from Necker-Enfants Malades Hospital and Cleveland Clinic. Informed consent was obtained from all subjects. This study was approved by local ethics committee of the Rockefeller University Hospital and Cleveland Clinic. The genotyping was conducted by RT-PCR using specific Act1-D10N probes^44^.

### Reagents

The following antibodies were from eBioscience: rat anti-mouse CD4 (1:400, RM4–5), rat anti-mouse CD44 (1:400, IM7), rat anti-mouse IFNγ (1:500, XMG1.2), rat anti-mouse CD8 (1:400, 53–6.7), rat anti-mouse lgM (1:400, 11/41), rat anti-mouse IL-4 (1:500, 11b11), rat anti-mouse IL-21 (1:500, mhalx21), hamster anti-mousePD1 (1:500, J43). The following antibodies were from Biolegend: rat anti-mouse IL17A (1:400, TC11-18H10), rat anti-mouse Bcl6 (1:400,7D1), mouse anti-mouse Fas (1:400, SA367H8), rat anti-mouse CD138 (1:400, 281–2), hamster anti-mouse CD3 (1:400, 145–2c11), rat anti-mouse B220 (1:600, RA3–6B2), rat anti-mouse CD45 (1:2000, 30–F11), hamster anti-mouse ICOS (1:400, C398.4A), rat anti-mouse CXCR5 (1:500, L138D7). The following antibodies were from BD: rat anti-mouse CD23 (1:500, B3B4), rat anti-mouse CD21 (1:500, 7G6), rat anti-mouse IL-4 (for cell culture,1:1000, 11B11, cat: 554432), rat anti-mouse IFNγ (for cell culture,1:1000, XMG1.2, cat: 559065), purified NA/LE hamster anti-mouse CD3e (1:500, 145–2C11, cat: 553057) and purified NA/LE hamster anti-mouse CD28 (1:500, 37.51, cat: 553294). Rabbit Anti-mouse/human STAT3 (1;1000, D3Z2G, cat: 12640S), Stat Antibody Sampler Kit II (1:1000, Cat: 93130), rabbit anti-mouse p-STAT1 (Tyr701) (1:1000, 58D6, Cat: 9167S), rabbit anti-mouse p-STAT5 (Tyr694) (1:1000, D47E7, Cat:4322T), rabbit anti-mouse p-STAT6 (Tyr641)(1:1000, D8S9Y, Cat: 56554S), rabbit anti-mouse/human Phospho-STAT3 (Tyr705) (1:1000, Cat: 9131L), rabbit anti-HA (1:1000, C29F4 cat: 3724S) and rabbit anti-flag (1:1000, cat: 2368s) were from Cell Signaling Technology. Mouse anti-HA (1:1000, clone HA-7), mouse anti-M2 (1:1000, clone M2) were from Sigma. Rat anti-mouse GL7 antibody (1:200, GL-7, Cat: 13-5902-81) was brought from Thermo Fisher. Goat anti-Peanut Agglutinin (PNA, 1:100, AS2074) was from Vector lab. Rabbit anti-mouse IL-12Rb1 (1:1000, Cat: 97813), rabbit anti-mouse p-STAT2 (Y690) (1:1000, Cat: 53132) and rabbit anti-mouse p-STAT4 (Y693)(1:1000, Cat: 28815) were from Abcam. Goat anti-mouse/human β-Actin was from Santa Cruze (1:1000, C-2). Recombinant mouse IL-6 protein (406-ML-005) and recombinant mouse IL-23 Protein (1887-ML-010) were from R&D. CFSE (1:2000, cat: C34554) was from Molecular Probes. Horse anti-mouse lgG (H+L) (for immunofluorescence staining, 1:300, cat: TI-2000) was from Vector laboratories, Inc. Rat anti-mouse TureBlot antibody (1:1000, eB144) and mouse anti-rabbit TureBlot antibody (1:1000, eB182).

### Cell surface and intracellular staining

For intracellular staining, single-cell suspensions obtained from the spleen, kidney, and submandibular gland of mice were cultured for 5 h with phorbol 12-myristate 13-acetate (PMA, 20 ng/ml, Sigma) plus ionomycin (500 ng/ml, Sigma). GolgiStop (1:500, BD Biosciences) was added during the final 2 h of incubation. Cells were stained with cell surface antibody for 30 min at 4 °C followed with fixation/permeabilization solution (BD Cytofix/Cytoperm™ Kit) according to the manufacturer’s instructions. After washing three times with Perm/Wash buffer, antibodies for intracellular staining were added for 1 h at 4 °C and cells were analyzed on a FACSCalibur (BD).

### Quantitative real-time PCR

Mice were perfused with 1× PBS. Kidneys and glands were harvested and homogenized with an OMNI TH tissue homogenizer (Omni International) and total RNA was extracted using TRIzol reagent according to the manufacturer’s instructions (Invitrogen). The cDNA was synthesized with Oligo (dT) primers and superscript II reverse transcriptase (Invitrogen) by using 1 μg total RNA. The cDNA was resuspended in 100 μl of H_2_O, and 2 μl of cDNA samples were used for real-time PCR in a total volume of 20 μl of SYBR Green reagent (Invitrogen) and specific primers. All gene expression results are expressed as arbitrary units relative to the expression of β-actin. The following RT-PCR primers ordered from Invitrogen were used for mouse genes: *Il21*, 5′-CCCTTGTCTGTCTGGTAGTCATC-3′ and 5′-ATCACAGGAAGGGCATTTAGC-3′; *Bcl2*, 5′-GGAAGGTAGTGTGTGTGG-3′ and 5′-ACTCCACTCTCTGGGTTCTTGG-3′; *Bclxl*, 5′-GCTGGGACACTTTTGTGGAT-3′ and 5′-TGTCTGGTCACTTCCGACTG-3′; *β-actin*, 5′- AGATGTGGATCAGCAAGCAG-3′ and 5′-GCGCAAGTTAGGTTTTGTCA-3′; *Il17a*, 5′-GTCCAGGGAGAGCTTCATCTG-3′ and 5′-CTTGGCCTCAGTGTTTGGAC-3′; *Sox5*, 5′-GAACAGCATAGGTCTCAGCCAC-3′ and 5′-CATGGCTAAATTTCCCTTCTTC-3′; *Rorγt*, 5′-CCATTCAGTATGTGGTGGAGTT-3′ and 5′-CTGACTAGGACGACTTCCATTG-3′.

### Histology and immunostaining

Tissues were immediately frozen in OTC embedding media and were sectioned or serially sectioned to obtain consecutive slices. Sections were stained with anti-lgG dsRed (H+L) and DAPI. All images were captured with a DP71 digital camera (Olympus) attached to an Olympus BX41 microscope.

### Antibodies and autoantibodies measurement

Serum IgM, IgG1, IgG2a, IgG2b, IgG3, IgA, Anti-histone IgG, anti-dsDNA, and anti-ssDNA were detected using ELISA kits. 96-well plates were coated with 50–100 µL capture antibodies for overnight at 4 °C or 2 h in room temperature following the manufacturer’s instruction. Wash wells four times using an automated 96-well plate washer and add 100 µL diluted serum or standards incubating at room temperature for 2 h. Wash wells four times and add 100 µL of diluted detection antibody to each well incubating for 1 h. After washing, add 100 µL of diluted HRP conjugate to each well for 30 min. Thoroughly aspirate and add 100 µL of substrate to each well and develop plate at room temperature in the dark for 30 min. Stop the reaction by adding 100 µL of stop solution. While the levels of Ig isotypes were read at 405 nm, the levels of autoantibodies were read at 450 nm. The antibody concentrations were calculated using Ig standards, provided by the manufacturer.

### Anti-IL21 antibody treatment in vivo

Anti-IL-21 antibody was obtained from Dr. Ken Coppieters, Principal Scientist in Novo Nordisk A/S. Act1-deficient were treated with anti-IL-21 neutralizing antibody intraperitoneally at 0.5 mg/mouse every other day for 3 months. Serum was collected monthly to assess the levels of autoantibodies (anti-histone lgG, anti-dsDNA, and anti-ssDNA) and immunoglobulin isotypes.

### Retroviral and lentiviral production

For infection of Naive T cells and B cells, viral supernatant were collected 36 h after transfection of Phoenix cells (ATCC, ATCC® Number: CRL-3213™) with 5 µg sequence encoding HA-tagged Act1 or Act1 mutant (∆300–309) cloned into pMSCV-pBabeMCS-IRES-RFP (Addgene plasmid # 33337). ATCC reported to use morphology, karyotyping, and PCR-based approaches to confirm the identity. Plasmocin was used to rule out the contamination during the cell culture. For knockdown Act1 gene in naive T cells, shRNA plasmids ordered from Sigma (TRCN0000105990 and TRCN0000105992) were transduced into Phoenix cells and viral supernatant were collected 36 h after transfection.

### In vitro T cell polarization

Cell suspensions from spleens were prepared as described before. For in vitro Th17 polarization, CD3^+^CD4^+^CD44^low^ naive T cells were sorted by flow cytometry and cultured in 24-well plates coated with anti-CD3 (1 µg/ml, BD) and anti-CD28 (2 µg/ml, BD) for 3 days. For Th0 polarization, anti-IFNγ (10 µg/ml) and anti-IL-4 (10 µg/ml) neutralizing antibodies were added to the culture. For Th17 polarization, anti-IFNγ (10 µg/ml) and anti-IL-4(10 µg/ml) neutralizing antibodies, IL-6 (10 ng/ml) and IL-23 (10 ng/ml) were added to the culture. To generate antigen-specific Th17 cells for Th17 and B cell co-culture, Naive T cells were isolated from MoG_35–55_ immunized mice and polarized in the presence of the cytokines above with additional MOG_35-55_ peptide and sorted dendritic cells which were also isolated from MOG_35–55_ immunized mice. The purity of Th17 cells were determined by flow cytometry in 3–5 days.

### B cell differentiation (CFSE)

B220^+^ cells were sorted by flow cytometry from spleen of MOG_35-55_ immunized WT and *Act1*^*−/−*^ mice and suspended in PBS (1 × 10^7^/ml). Subsequently, 2× CFSE dye (Molecular Probe) was added to a final concentration of 2.5 µM/ml. After incubation for 7 min at room temperature, cells were washed with cold DMEM media with 10% FBS for three times and co-cultured with Th0, Th17 cells plus MOG_35–55_ peptide (2 ng/ml), anti-IFNγ (10 µg/ml), anti-IL-4 (10 µg/ml) with and without anti-IL21 neutralization antibody (10 µg/ml). On day 3, cells were stained with antibodies against cell surface markers CD4 and B220 followed by flow cytometry analysis.

### Th17 induced antibody production in vitro

Sorted B220^+^ cells from spleens of WT, *Act1*^*−/−*^, *Il21r*^*−/−*^, and *Act1*^*−/−*^*Il21r*^*−/−*^ mice (C57BL/6 background) were co-cultured with Th0 or differentiated Th17 cells in U-bottom 96 plates plus MOG_35-55_ peptide (2 ng/ml), anti-IFNγ (10 µg/ml) and anti-IL-4(10 µg/ml) for 10 days. The supernatants of the culture were analyzed for immunoglobulin isotypes.

### Immunoblotting and immunoprecipitation

HeLa cell line (ATCC® CCL-2™) was brought from ATCC and it reported to use morphology, karyotyping, and PCR-based approaches to confirm the identity. Plasmocin was used to rule out the contamination during the cell culture. Cells were lysed in lysis buffer (0.5% Triton X-100, 20 mM HEPES, pH 7.4, 150 mM NaCl, 12.5 mM β-glycerophosphate, 1.5 mM MgCl_2_, 10 mM NaF, 2 mM dithiothreitol, 1 mM sodium orthovanadate, 2 mM EGTA, 20 mM aprotinin, 1 mM phenylmethylsulfonyl fluoride). Twenty micrograms of protein lysate was run per lane on a 12% SDS-PAGE gel and subjected to immunoblotting with different antibodies. Co-immunoprecipitation experiments were performed as described previously^3^.

### Proximity ligation assay

Direct Act1 and STAT3 protein interaction was assessed by a proximity ligation assay following the instructions of Duolink in Situ Fluorescence Kit (Sigma). HeLa cells were transfected with V5-tagged Act1 and Flag-tagged stat3 plasmids for 3 days and washed with cold washing buffer followed by fixation at room temperature for 5 min in 2% paraformaldehyde. After washing three times, blocking buffer containing 0.1% Triton X-100, 5% goat serum and 3% H_2_O_2_ was performed to allow permeabilization at 37 °C for 1 h. Cells were further incubated with primary antibodies (mouse anti-V5, rabbit anti-FLAG; Sigma), secondary antibodies (anti-rabbit PLUS and anti-mouse MINUS) and green detection reagent. The steps of incubation, ligation, and amplification were all performed following manufacturer instructions.

### Statistical analysis

Two-tailed Student’s *T* test was performed to determine the significance of differences between two groups. All results were demonstrated as mean ± SEM. *p < 0.05; **p < 0.01; ***p < 0.001. All data were from at least two independent experiments.

### Data availability

All relevant data are available from the authors.

## Electronic supplementary material

Supplementary Information
