# Supplementary material for: Act1 is a negative regulator in T and B cells via direct inhibition of STAT3
Source: Nat Commun. 2018 Jul 16;9:2745. doi: 10.1038/s41467-018-04974-3 (PMC6048100; doi:10.1038/s41467-018-04974-3)
Supplement: Supplementary file 1 — Supplementary Information [file 41467_2018_4974_MOESM1_ESM.pdf]

Supplementary Information

Act1 is a negative regulator in T and B cells via direct inhibition of STAT3

Zhang, et al.

Supplementary Figures

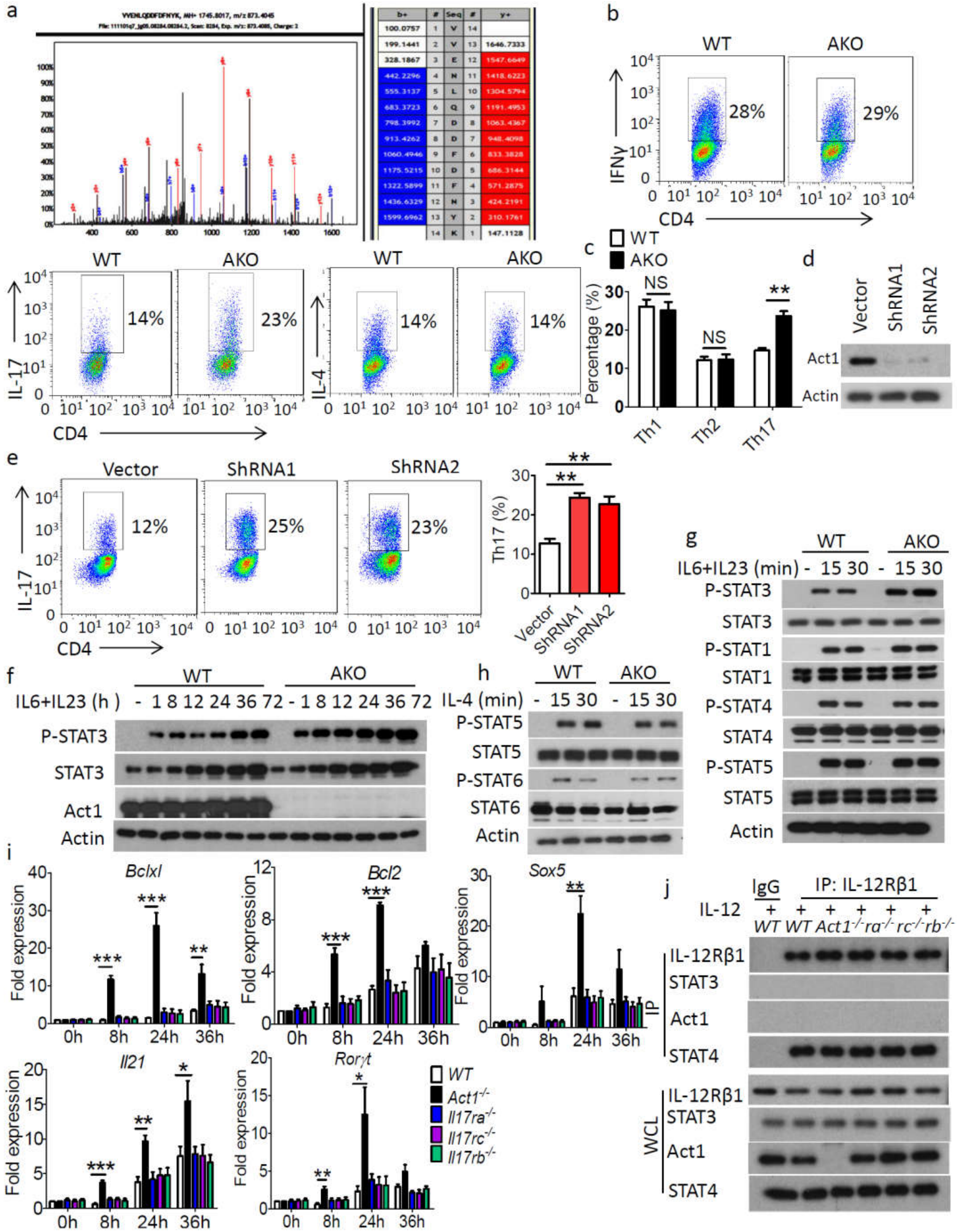

Supplementary Figure 1. Act1 physically interacts with STAT3 and negatively regulates STAT3 activation (**a**) Mass spectrometry analysis of Act1-associated proteins after immunoprecipitation via anti-Flag beads from lysates of Hela cells transiently transfected to express Act1-Flag. One of fifteen matched peptide sequences that correspond to STAT3 was shown as a sample. In peptide sequences, colored lines on the peptide backbone correspond to b- and y-type ion series observed in the spectra. **(b-c)** Naive $CD4^+$ T cells sorted from spleen of WT and *Act1*$^{-/-}$ mice were polarized to Th1, Th2, Th17 cells with IL-12, IL-4 or IL-6+23 for three days, respectively (b). The level of cells was quantified as percentage of total $CD4^+$ T cells (c). **(d-e)** Western blot analysis of Act1 knockdown levels in naive $CD4^+$ T cells after transfection with shRNA or vector control (d). Naive $CD4^+$ T cells with or without Act1 knockdown were polarized with IL-6+23 for 3 days, followed by flow analysis of Th17 cells ($CD3^+CD4^+IL\text{-}17^+$). The levels of Th17 cells were presented as percentage of total $CD4^+$ T cells (e). (**f-h**) Naive $CD4^+$ T cells were stimulated with IL-6+23 (f-g) or IL-4 (h) for the indicated times, followed by western analysis with the indicated antibodies. (**i**) RT-PCR analysis of STAT3 target genes in Naïve T cells from WT, *Act1*$^{-/-}$, *Il17ra*$^{-/-}$, *Il17rc*$^{-/-}$ and *Il17rb*$^{-/-}$ mice stimulated with IL-6 for the indicated times. (**j**) Naive T cells from WT, *Act1*$^{-/-}$, *Il17ra*$^{-/-}$, *Il17rc*$^{-/-}$and *Il17rb*$^{-/-}$ mice were stimulated with IL-12 for 1 hour, followed by immunoprecipitation with anti-IL12Rβ1 and western analysis with the indicted antibodies. N=4~6/group. Mean±SEM. *P<0.05; **P<0.01; ***P<0.001. Two-tailed Student's T test. All the data presented were from two independent experiments.

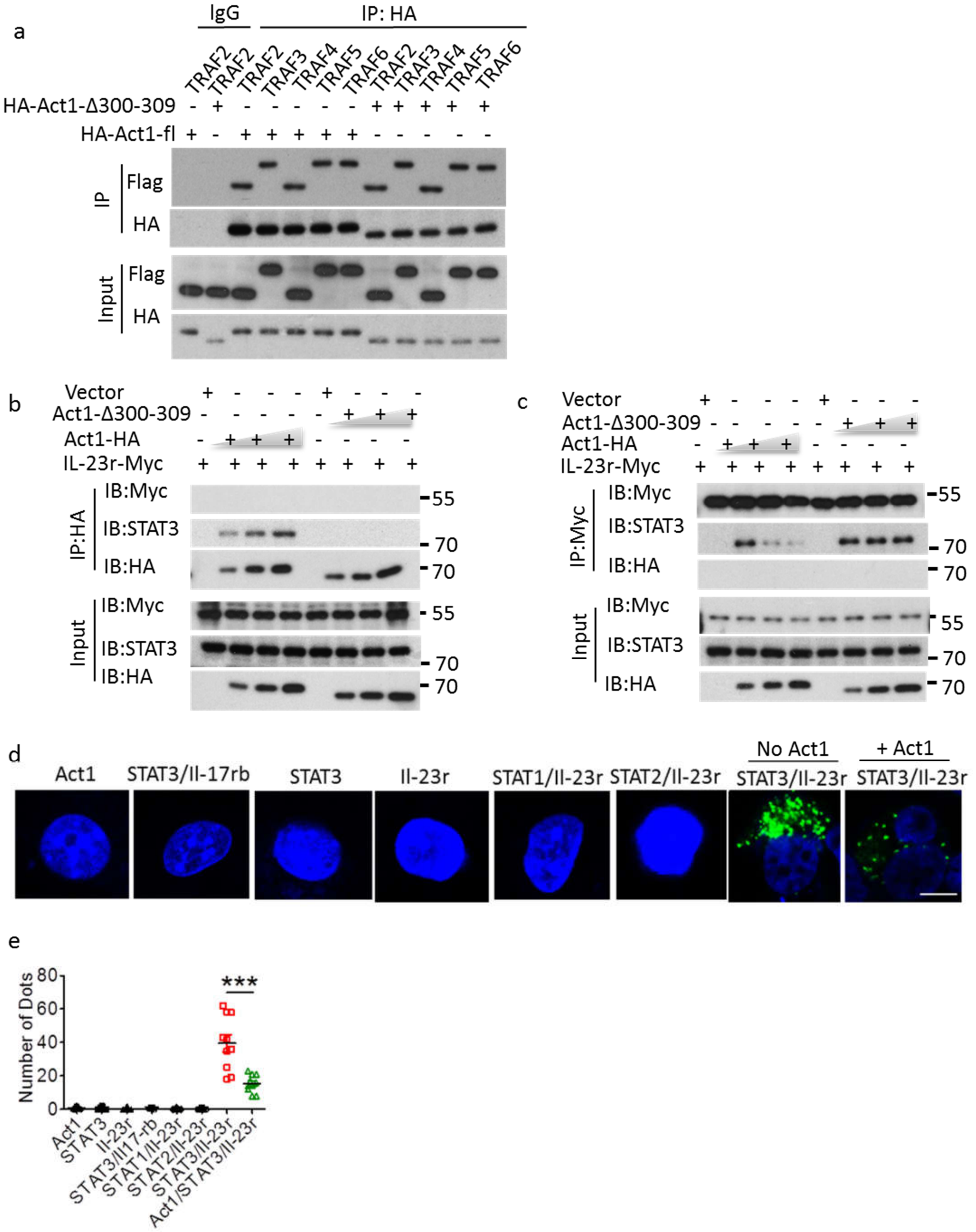
a
IgG
IP: HA
TRAF2
TRAF2
TRAF2
TRAF3
TRAF4
TRAF5
TRAF6
TRAF2
TRAF3
TRAF4
TRAF5
TRAF6
HA-Act1-Δ300-309
HA-Act1-fl
IP
Flag
HA
Input
Flag
HA
b
Vector
Act1-Δ300-309
Act1-HA
IL-23r-Myc
IP:HA
IB:Myc
IB:STAT3
IB:HA
Input
IB:Myc
IB:STAT3
IB:HA
55
70
70
55
70
70
c
Vector
Act1-Δ300-309
Act1-HA
IL-23r-Myc
IP:Myc
IB:Myc
IB:STAT3
IB:HA
Input
IB:Myc
IB:STAT3
IB:HA
55
70
70
55
70
70
d
Act1
STAT3/Il-17rb
STAT3
Il-23r
STAT1/Il-23r
STAT2/Il-23r
No Act1
STAT3/Il-23r
+ Act1
STAT3/Il-23r
e
Number of Dots
80
60
40
20
0
***
Act1
STAT3
Il-23r
STAT3/Il17-rb
STAT1/Il-23r
STAT2/Il-23r
STAT3/Il-23r
Act1/STAT3/Il-23r

Supplementary Figure 2. Act1 competes with IL-23R for STAT3 binding. (**a**) Hela cells were transiently co-transfected Flag-tagged TRAFs (TRAF2, TRAF3, TRAF4, TRAF5 and TRAF6) with full-length HA-tagged Act1 or Act1 mutant (Δ300-309). Lysates from the transfected cells were immunoprecipitated with anti-HA (HA-Act1), followed by western analysis with the indicated antibodies. **(b-c)** IL-23R was co-transfected into Hela cells with vector, increased amounts of Flag-tagged full-length Act1 and Act1 mutants. With IL-23 stimulation for 20 mins, lysates from transfected cells were immunoprecipitated with anti-Myc (IL-23R-Myc), or anti-Flag (M2), followed by analysis with the indicated antibodies. (**d-e**) Proximal ligation assay to examine the impact of Act1 on the interaction of STAT3 with IL-23R. Green dots present the interaction of STAT3 and *Il-23r*. Scale bars, 10μm. All the data presented were from two independent experiments.

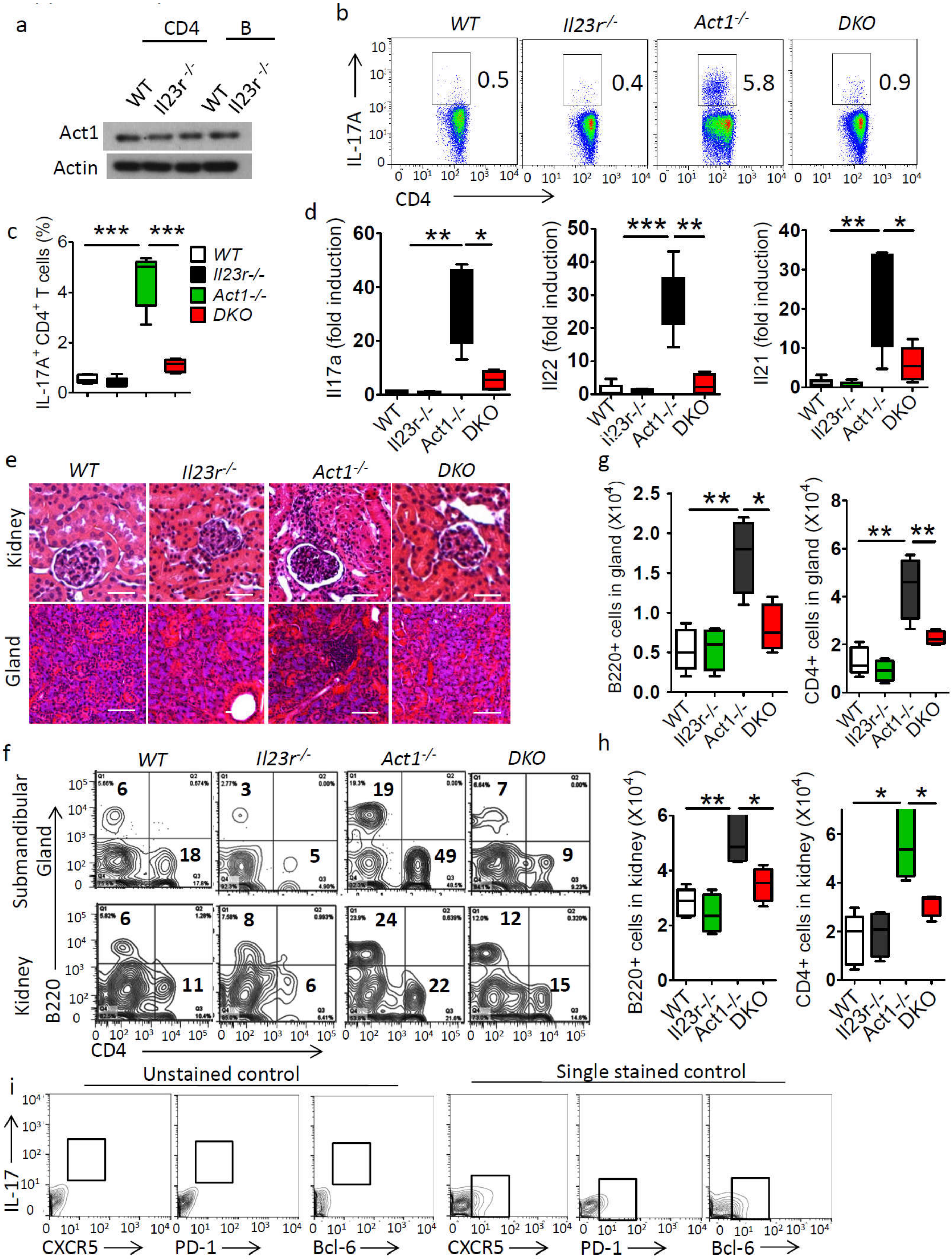
a
CD4
B
WT
Il23r -/-
WT
Il23r -/-
Act1
Actin
b
WT
Il23r-/-
Act1-/-
DKO
IL-17A
CD4
0.5
0.4
5.8
0.9
c
IL-17A+ CD4+ T cells (%)
***
***
WT
Il23r-/-
Act1-/-
DKO
d
Il17a (fold induction)
Il22 (fold induction)
Il21 (fold induction)
**
*
***
**
**
*
WT
Il23r-/-
Act1-/-
DKO
e
WT
Il23r-/-
Act1-/-
DKO
Kidney
Gland
g
B220+ cells in gland (X10^4)
CD4+ cells in gland (X10^4)
f
WT
Il23r-/-
Act1-/-
DKO
Submandibular Gland
Kidney
B220
CD4
6
18
3
5
19
49
7
9
6
11
8
6
24
22
12
15
h
B220+ cells in kidney (X10^4)
CD4+ cells in kidney (X10^4)
i
Unstained control
Single stained control
IL-17
CXCR5
PD-1
Bcl-6
CXCR5
PD-1
Bcl-6

Supplementary Figure 3. IL-23R is required for the autoimmune diseases in Act1$^{-/-}$ mice (a) Naive CD4^{+} T cells and B cells were sorted from spleens of WT and Il23r$^{-/-}$ mice, followed by western analysis for Act1 expression. (**b-c**) Flow cytometry analysis of IL-17A^{+} CD3^{+} CD4^{+} T cells isolated from spleen of 8-month-old mice (a) and the percentage of IL-17A^{+} CD3^{+} CD4^{+} T cell was indicated (b). (**d**) RT-PCR analysis of *Il17a*, *Il22* and *Il21* transcripts in spleen of 8-month-old mice. (**e**) Frozen section of kidney and gland from 8-month-old mice were stained with H&E. Scale bars, 50µm. (**f-h**) Flow cytometry analysis of CD45^{+}B220^{+} cells and CD45^{+}CD4^{+} cells from kidney and submandibular gland (F) and quantification of CD45^{+}B220^{+} cells and CD45^{+}CD4^{+} cells from submandibular gland and kidney (h). (**i**) Negative controls for Tfh cells in Fig 3D. N=4~6/group. Mean±SEM. *$P<0.05$; **$P<0.01$; ***$P<0.001$. Two-tailed Student's T test. All the data presented were from two independent experiments.

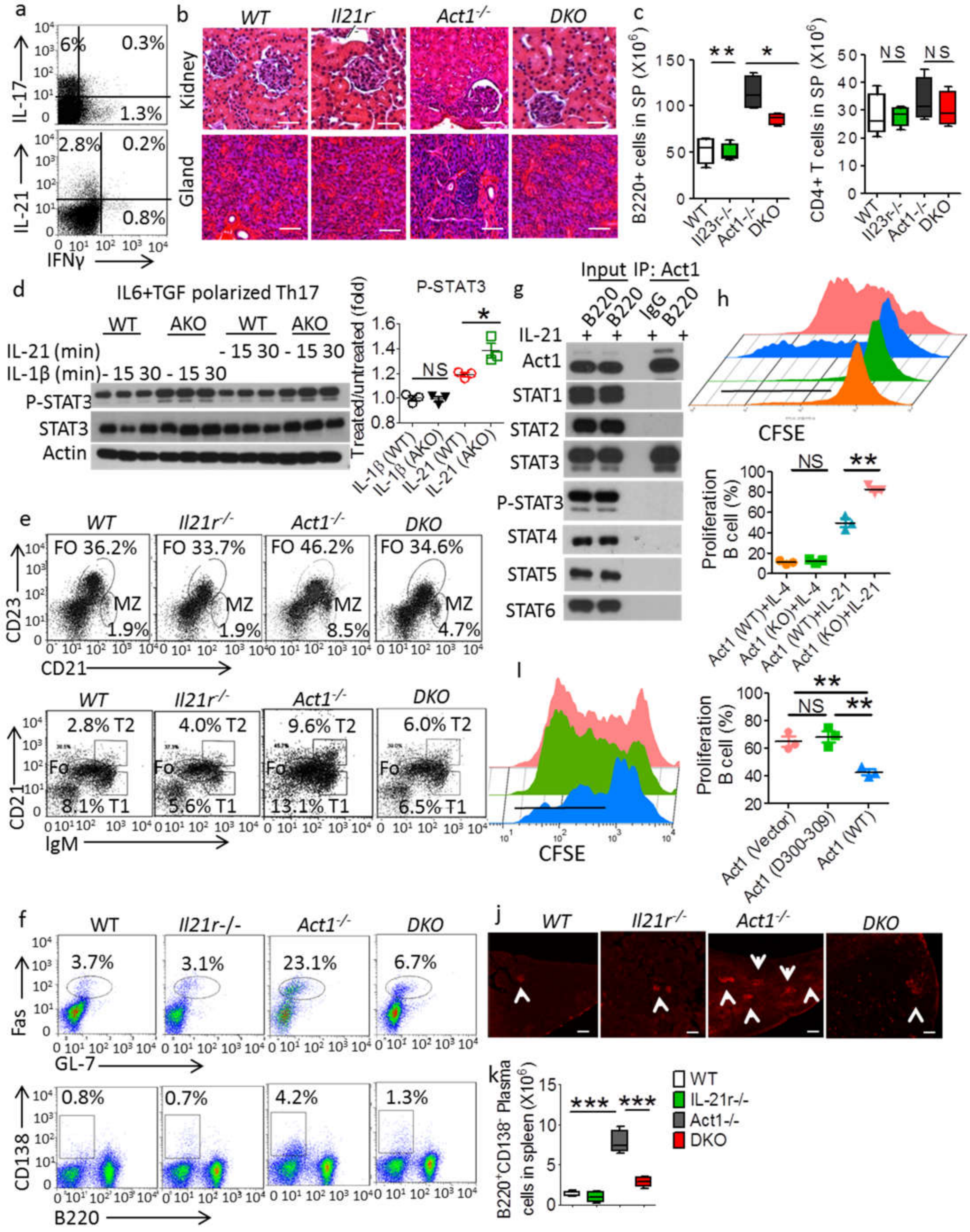

Supplementary Figure 4. IL-21R is required for the autoimmune diseases in Act1$^{-/-}$ mice (**a**) Flow cytometry analysis of IFNγ expression in IL-17^{+} or IL-21^{+} CD4^{+} T cells from spleens of 8-month-old *Act1$^{-/-}$* mice. (**b**) Frozen section of kidney and gland were stained with H&E. (**c**) Quantification of total CD45^{+}B220^{+} cells and CD45^{+}CD4^{+} cells in spleen of 8-month-old mice by flow cytometry. (**d**) Naive CD4^{+} T cells sorted from spleens of wild-type and *Act1$^{-/-}$* mice were polarized to Th17 cells with IL-6+TGFβ for three days, followed by IL-1β or IL-21 stimulation for 15 or 30 mins. The lysates were subjected to western analysis with the indicated antibodies. The phosphorylation of STAT3 were quantified as a ratio of treated (30 mins)-to-untreated samples. (**e-f**) Gating strategy of marginal zone B cells (B220^{+}CD21^{hi}CD23^{lo}), follicular B cells (B220^{+}CD21^{int}CD23^{hi}), T1 transitional B cells (B220^{+}IgM^{hi}CD21^{-}), T2 transitional B cells (B220^{+}IgM^{hi}CD21^{+}) (e); Germinal center B cells (B220^{+}GL7^{+}Fas^{+}) and plasma cells (B220^{-}CD138^{+}) from spleen of wild-type, *Act1$^{-/-}$*, *Il21r$^{-/-}$* and *Act1$^{-/-}$ Il21r$^{-/-}$* mice (f). (**g**) B220^{+} B cells isolated from spleens were stimulated with IL-21, followed by immunopricipitation with anti-Act1 and western analysis with the indicated antibodies. (**h**) Proliferation assay of wild-type and *Act1$^{-/-}$* B cells isolated from spleens were performed by labeling with CFSE followed by stimulation with IL-4 or IL-21. (**i**) WT-Act1 and Act1Δ300-309 mutant in retroviral vector (carrying RPF) were introduced into the primary *Act1$^{-/-}$* B cells. Sorted RFP^{+} B cells were labelled with CFSE and stimulated with IL-21 to measure cell proliferation. (**j-k**) 6-week-old WT and *Act1$^{-/-}$* mice were immunized intraperitoneally with 20μg NP-CGG. On day 14, the spleens were collected and quick frozen in OCT followed by immunofluorescence staining for PNA (j). The plasma cells in spleens were analyzed by flow cytometry (k). N=3~5/group. Mean±SEM. Scale bars, 50μm. *P<0.05; **P<0.01; Two-tailed Student's T test. All the data presented were from two independent experiments.

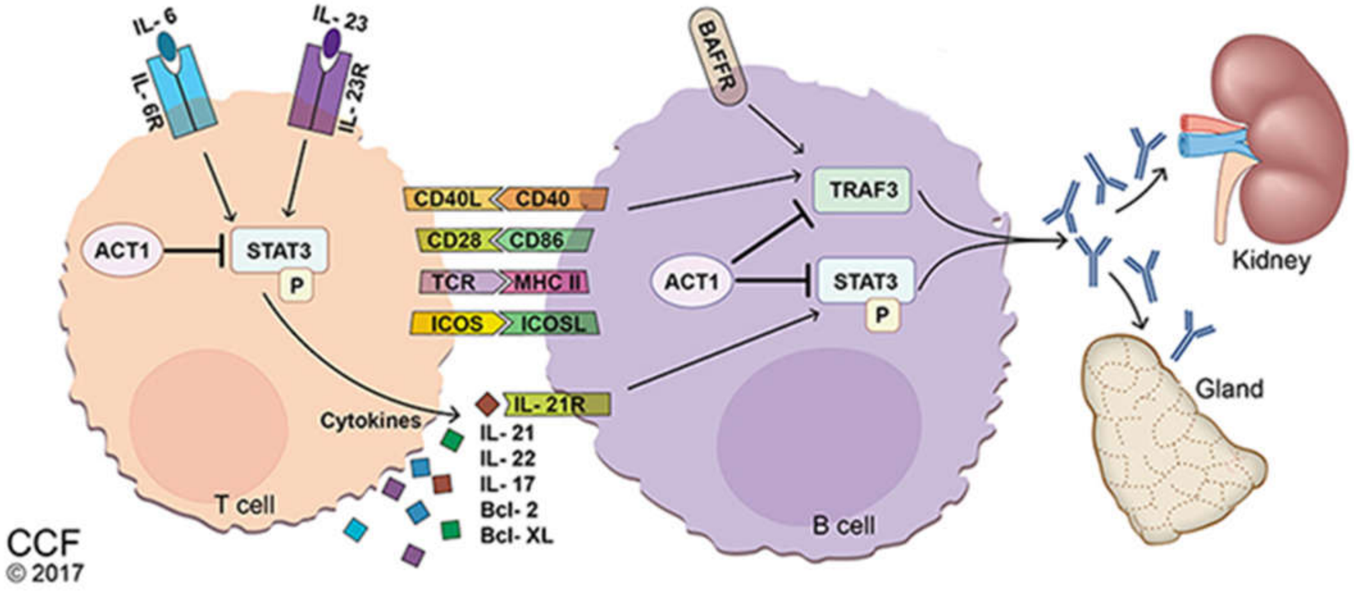

Supplementary Figure 5. Model of Act1 deficiency associated autoimmunity. Act1 forms a complex with STAT3, preventing STAT3's recruitment to IL-23R and IL-21R and attenuating STAT3 activation in Th17 and B cells, respectively. Act1 also interacts with TRAF3, which leads to inhibition of CD40/BAFFR signaling in B cells[1]. Deficiency of Act1 results in hyper activated Th17 cells with elevated IL-21 expression, which in turn promotes T-B cell interaction for B cell expansion and antibody production contributing to autoimmunity. Reprinted with permission, Cleveland Clinic Center for Medical Art & Photography © 2018. All Rights Reserved.

Supplementary Figure 6. Gating strategy

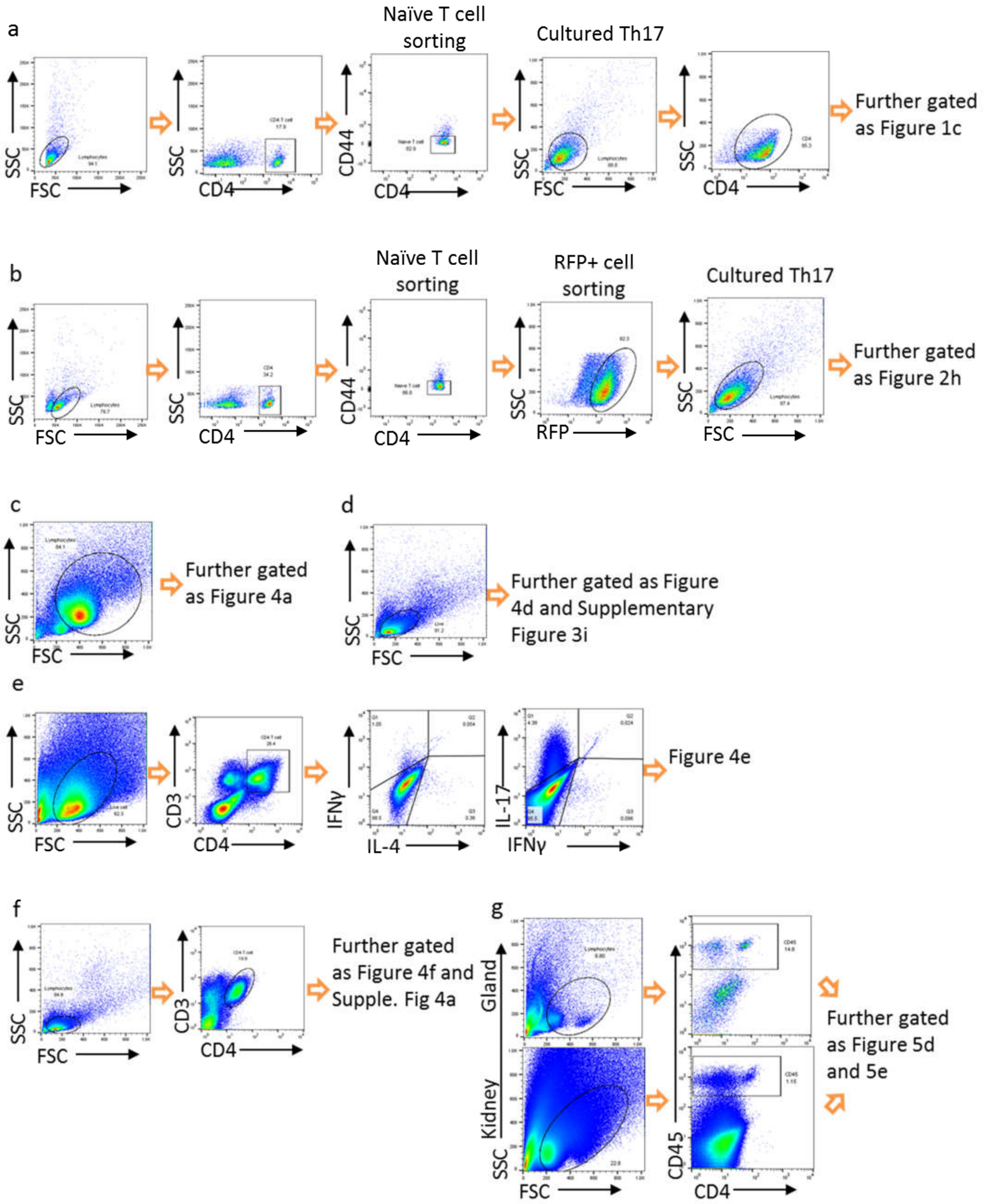

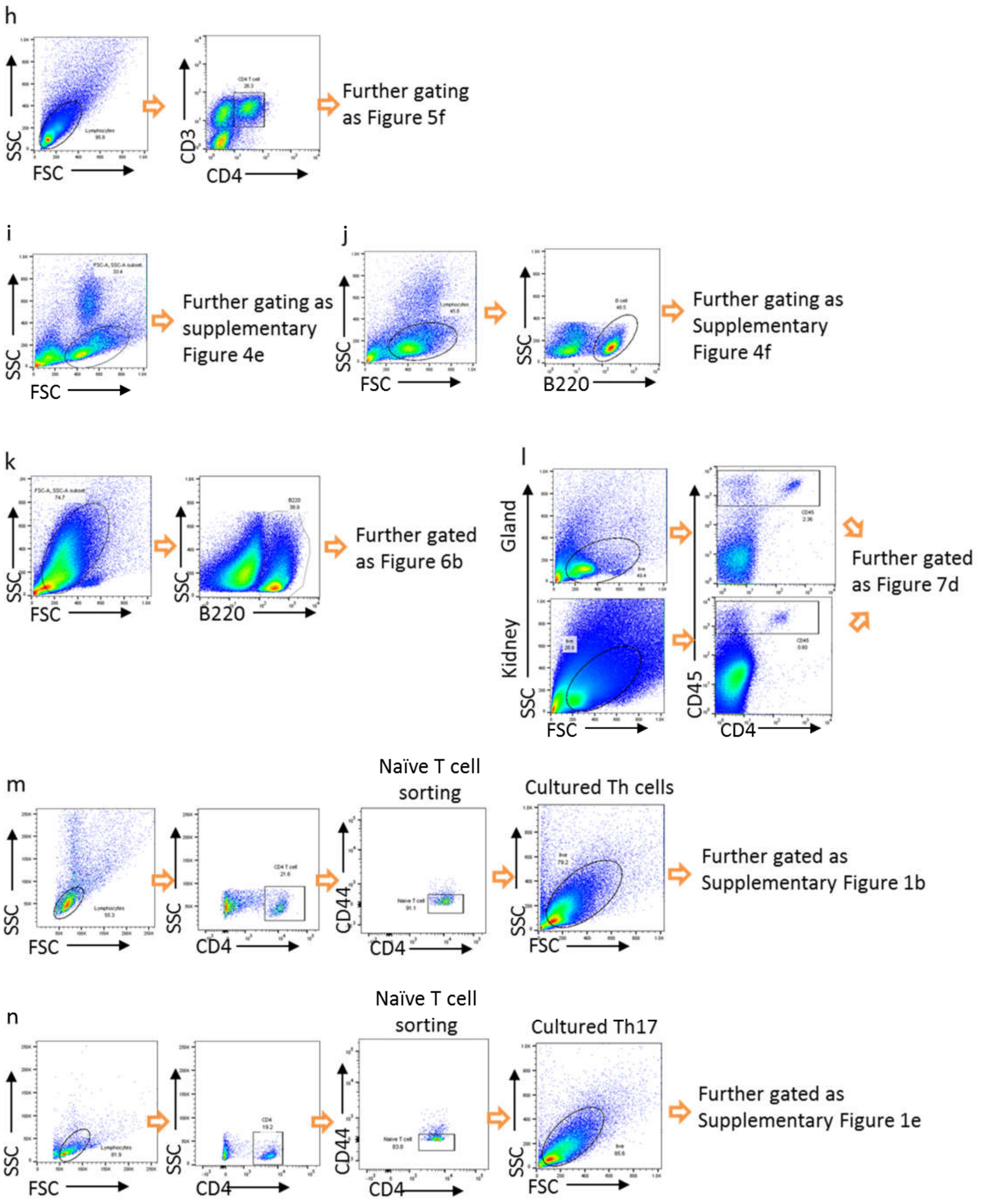
h
SSC
FSC
CD3
CD4
Further gating as Figure 5f
i
Further gating as supplementary Figure 4e
j
B220
Further gating as Supplementary Figure 4f
k
Further gated as Figure 6b
l
Gland
Kidney
CD45
Further gated as Figure 7d
m
Naïve T cell sorting
Cultured Th cells
CD44
Further gated as Supplementary Figure 1b
n
Cultured Th17
Further gated as Supplementary Figure 1e

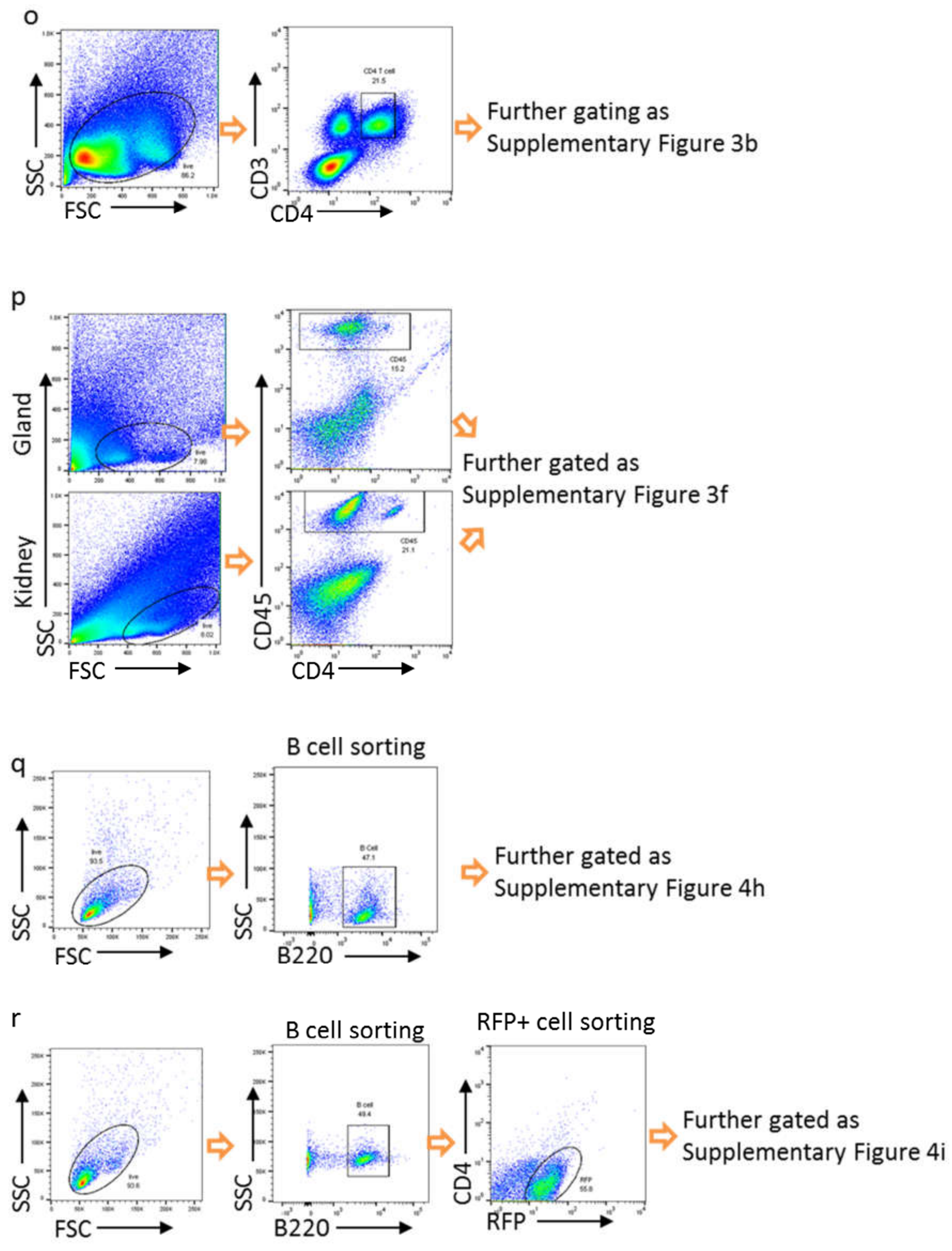

Supplementary Figure 6. Schematic gating strategy of flow cytometry. (**a**) Relates to Fig. 1c. (**b**) Relates to Fig. 2h. (**c**) Relates to Fig. 4a. (**d**) Relates to Fig. 4d and Supplementary Fig. 3i. (**e**) Relates to Fig. 4e. (**f**) Relates to Fig. 4f and Supplementary Fig. 4a. (**g**) Relates to Fig. 5d and 5e. (**h**) Relates to Fig. 5f. (**i**) Relates to Fig. 6a and Supplementary Fig. 4e. (**j**) Relates to Fig. 6a and Supplementary Fig. 4f. (**k**) Relates to Fig. 6b. (**l**) Relates to Fig. 7d. (**m**) Relates to Supplementary Fig. 1b. (**n**) Relates to Supplementary Fig. 1e. (**o**) Relates to Supplementary Fig. 3b. (**p**) Relates to Supplementary Fig. 3f. (**q**) Relates to Supplementary Fig. 4h. (**r**) Relates to Supplementary Fig. 4i.

Supplementary Figure 7. Uncropped images of films for all the figures

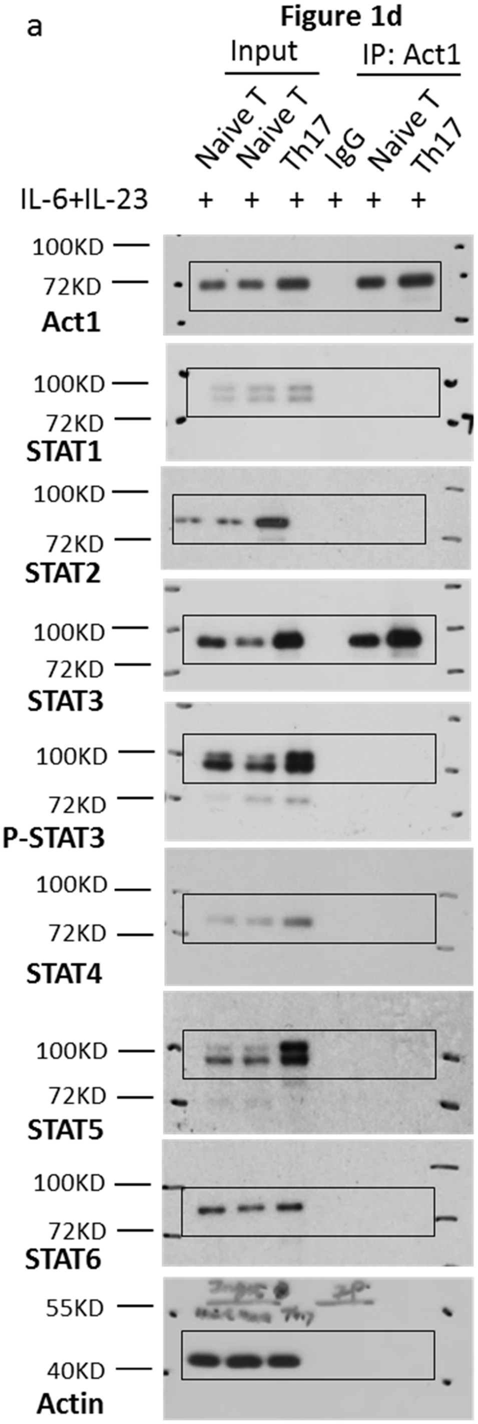

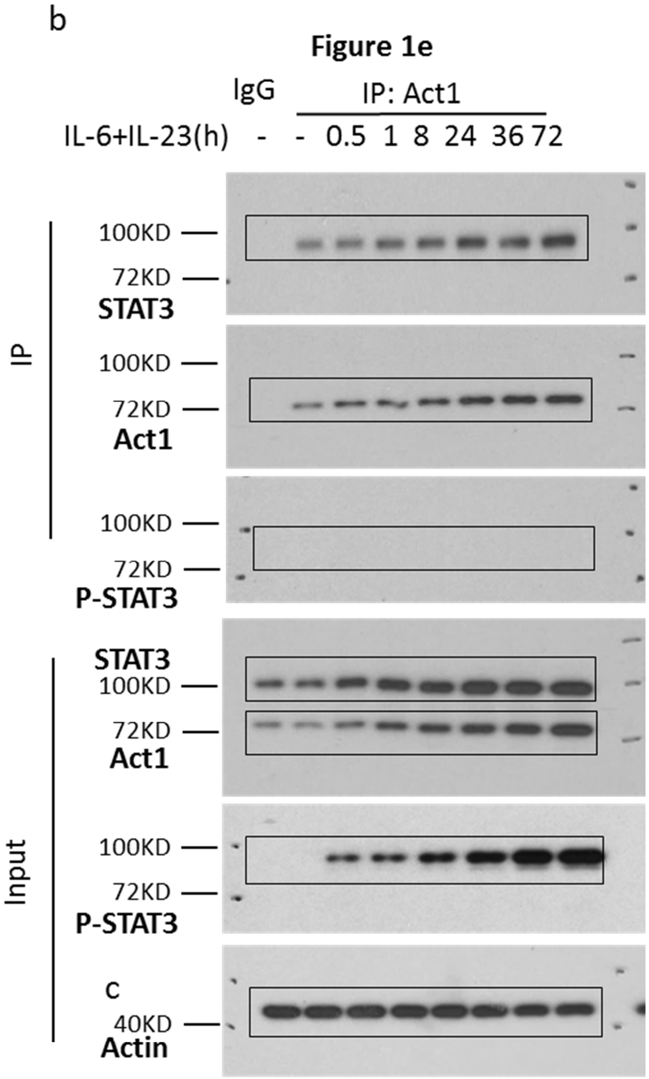

c

Figure 2a

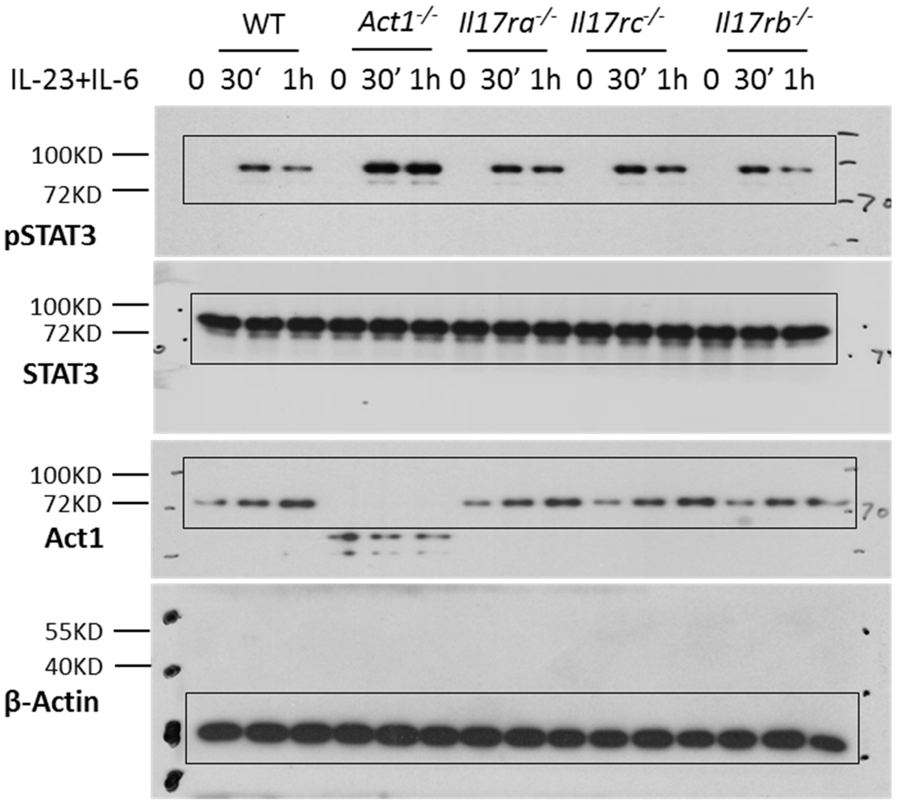

d

Figure 2c

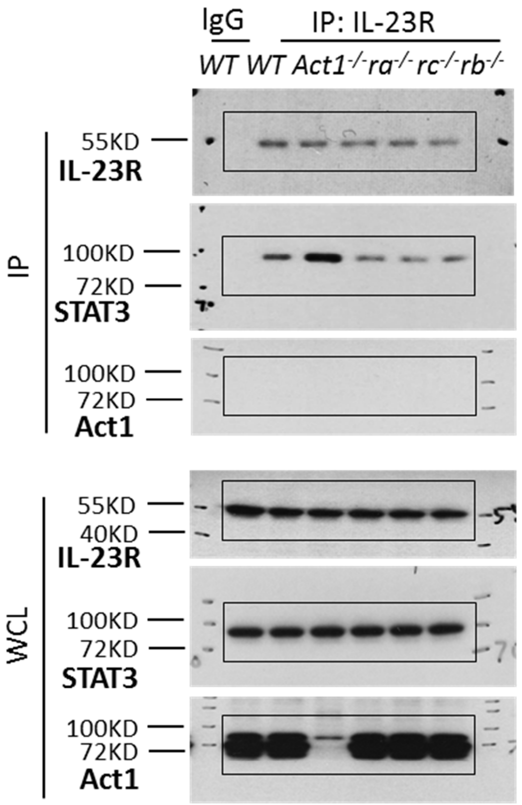

e

Figure 2e

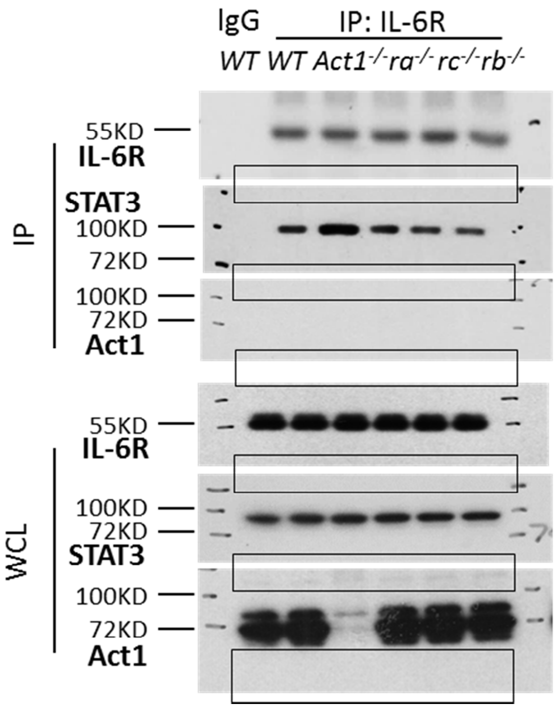

f

Figure 2g

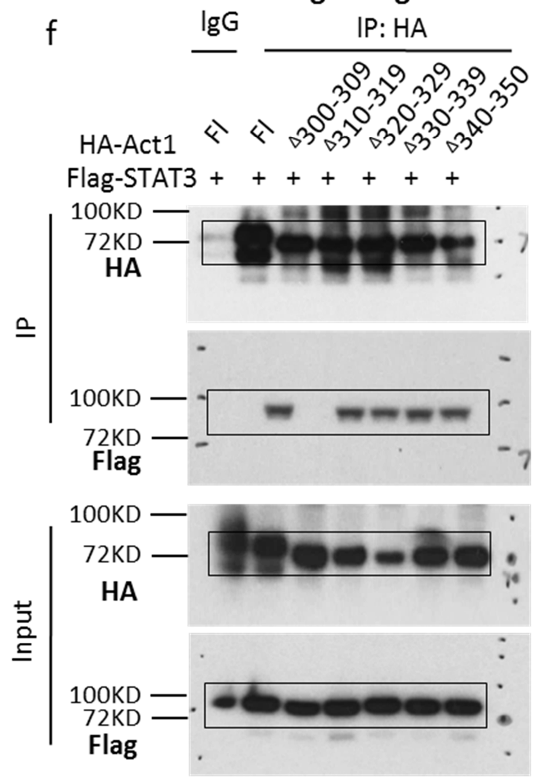

g

Figure 2i

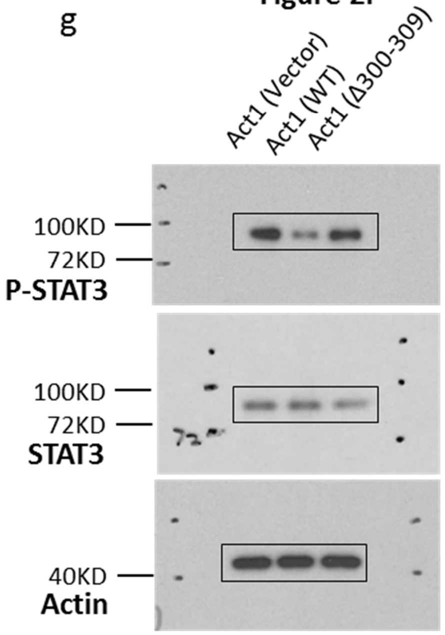

h

Figure 6d

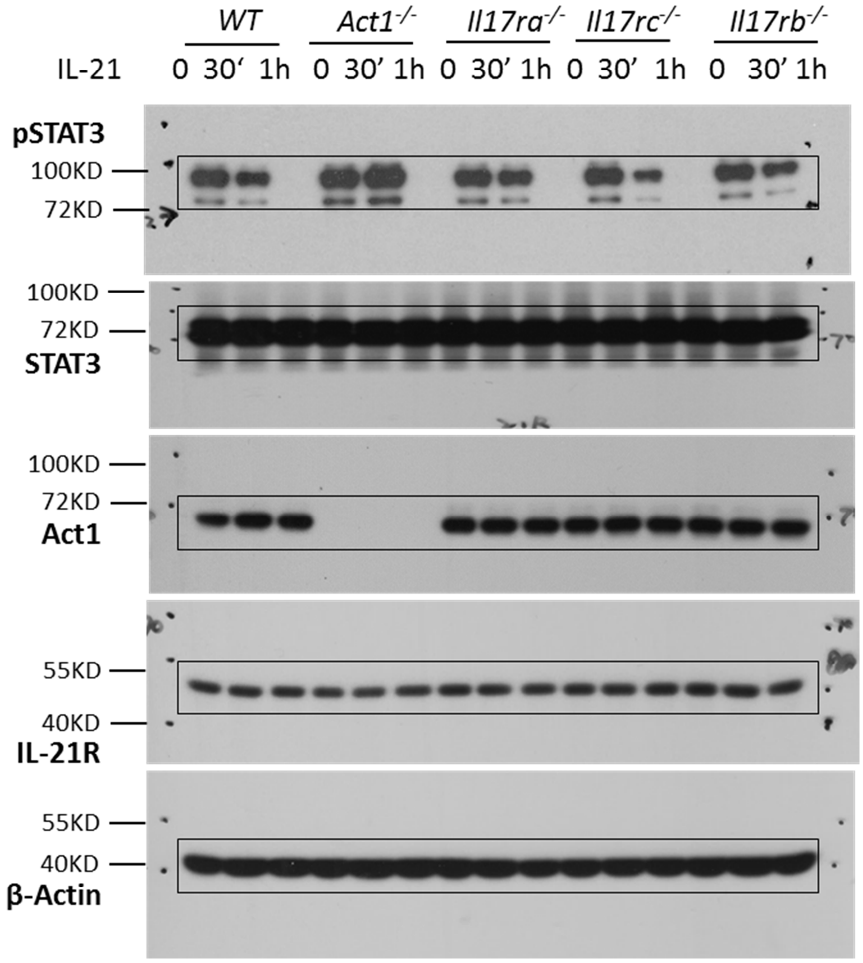

Figure 6e

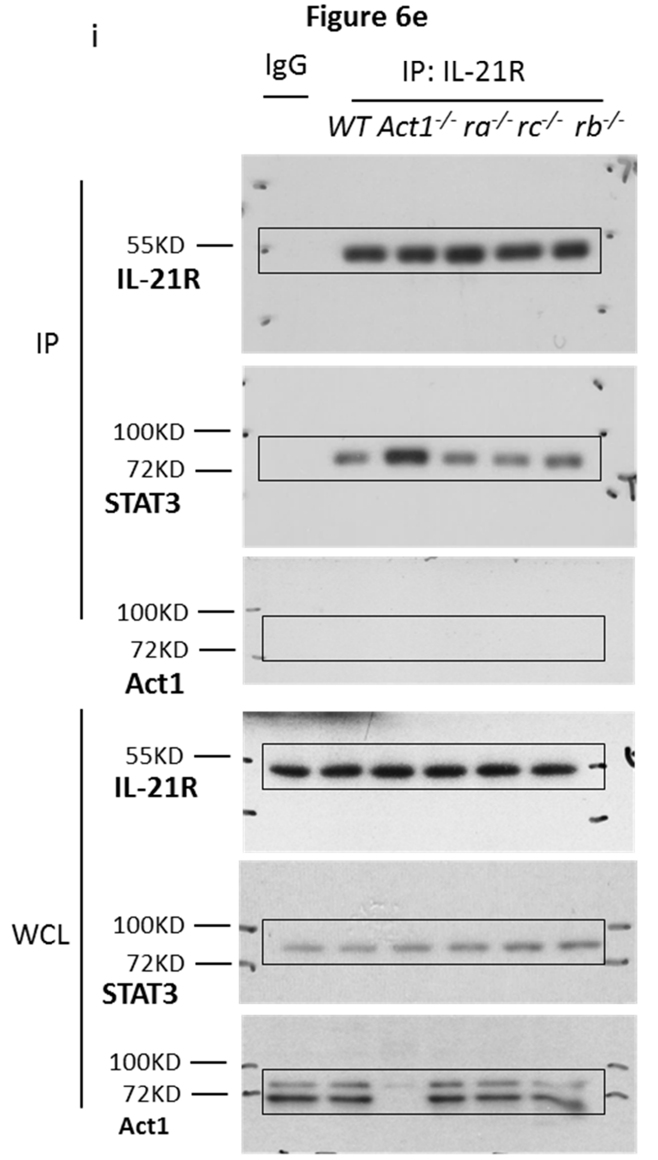

Figure 7g

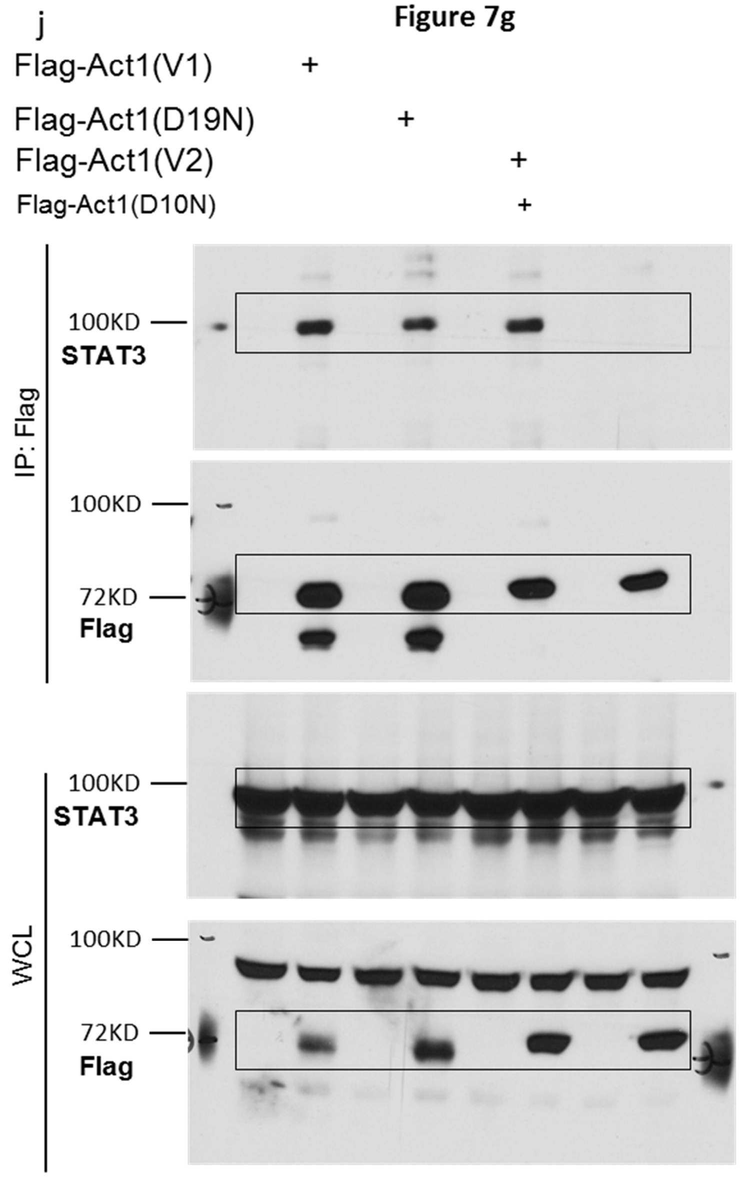

Figure 7h (Left panel)

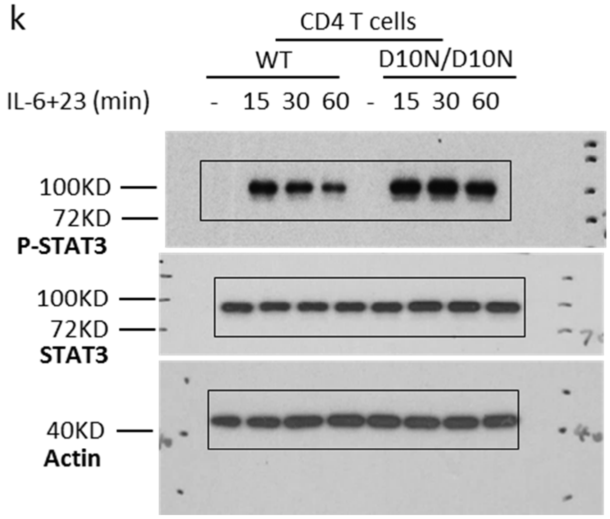

Figure 7h (Right panel)

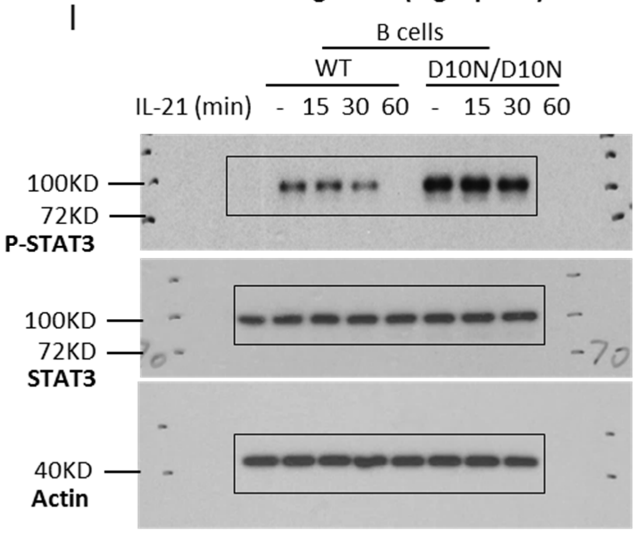

m

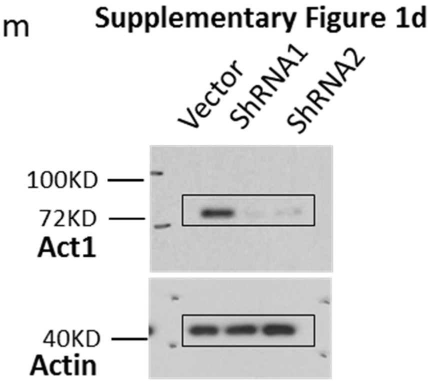
Supplementary Figure 1d
Vector
ShRNA1
ShRNA2
100KD
72KD
Act1
40KD
Actin

n

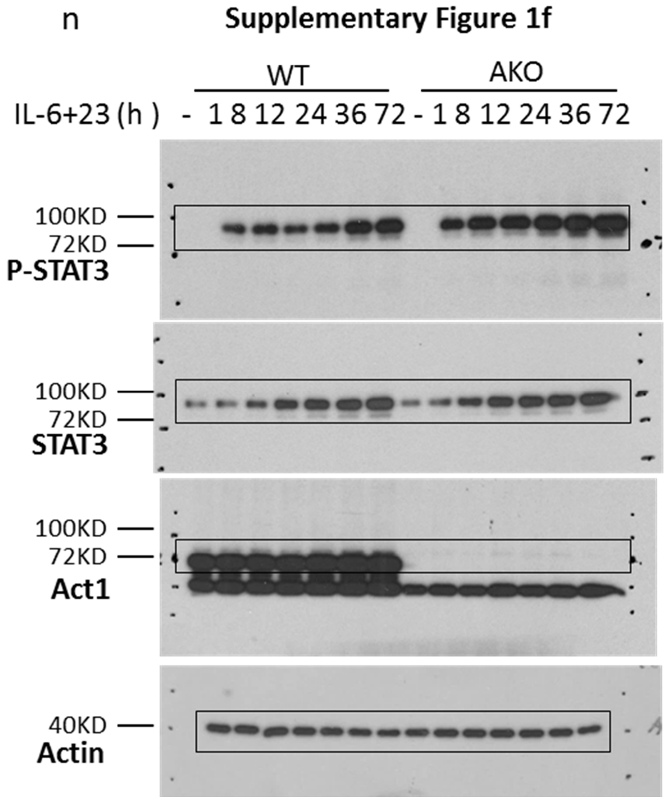
Supplementary Figure 1f
WT
AKO
IL-6+23 (h) - 1 8 12 24 36 72 - 1 8 12 24 36 72
100KD
72KD
P-STAT3
100KD
72KD
STAT3
100KD
72KD
Act1
40KD
Actin

o **Supplementary Figure 1g**

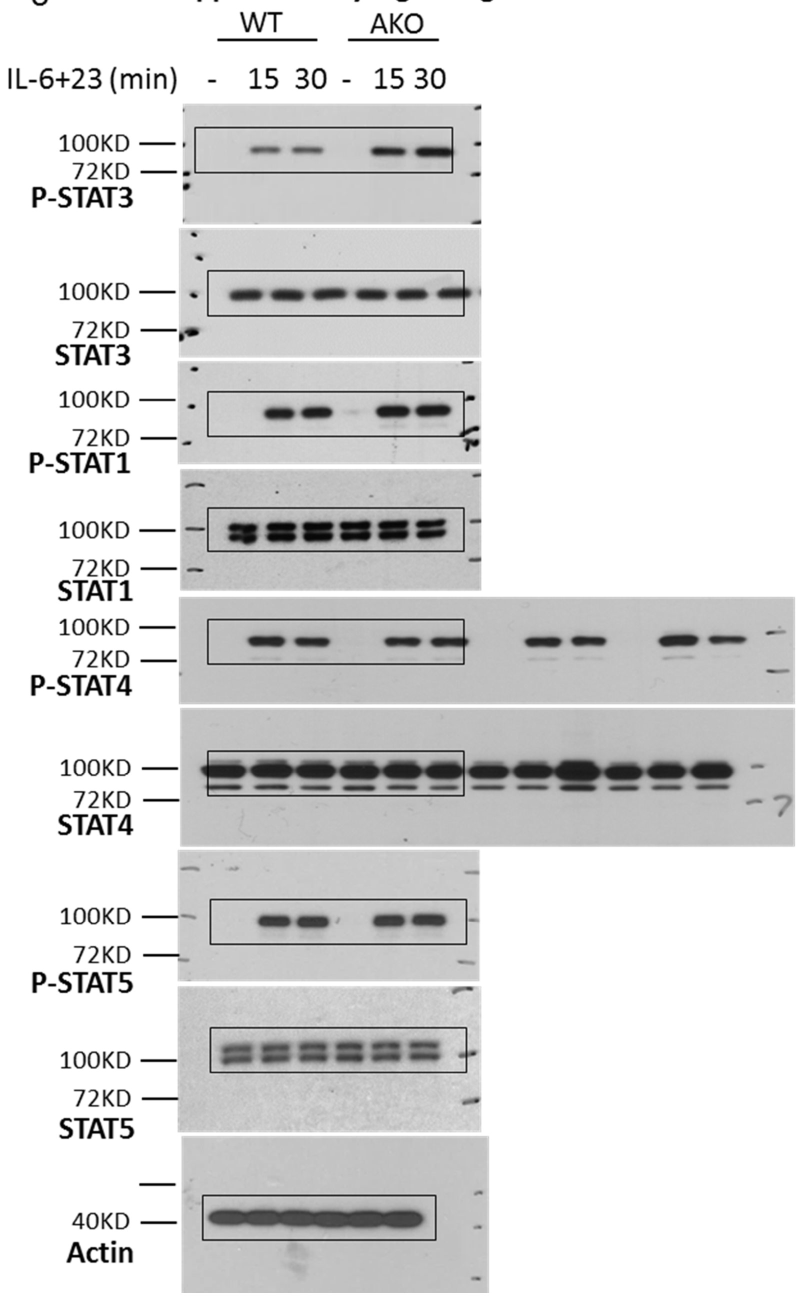

p **Supplementary Figure 1h**

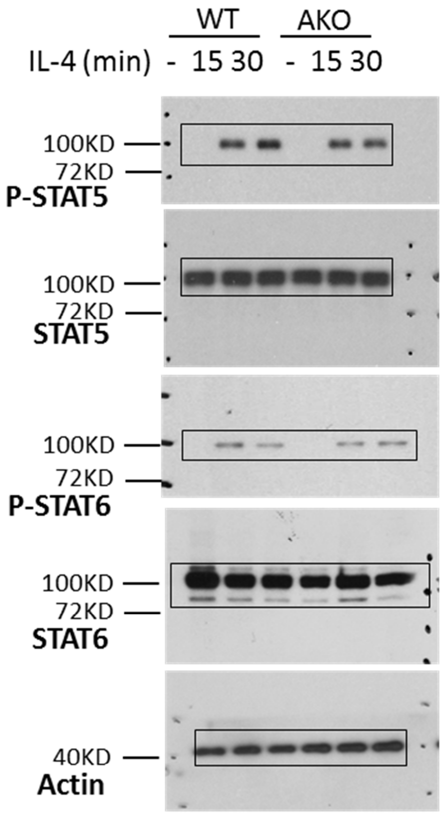

q

Supplementary Figure 1j

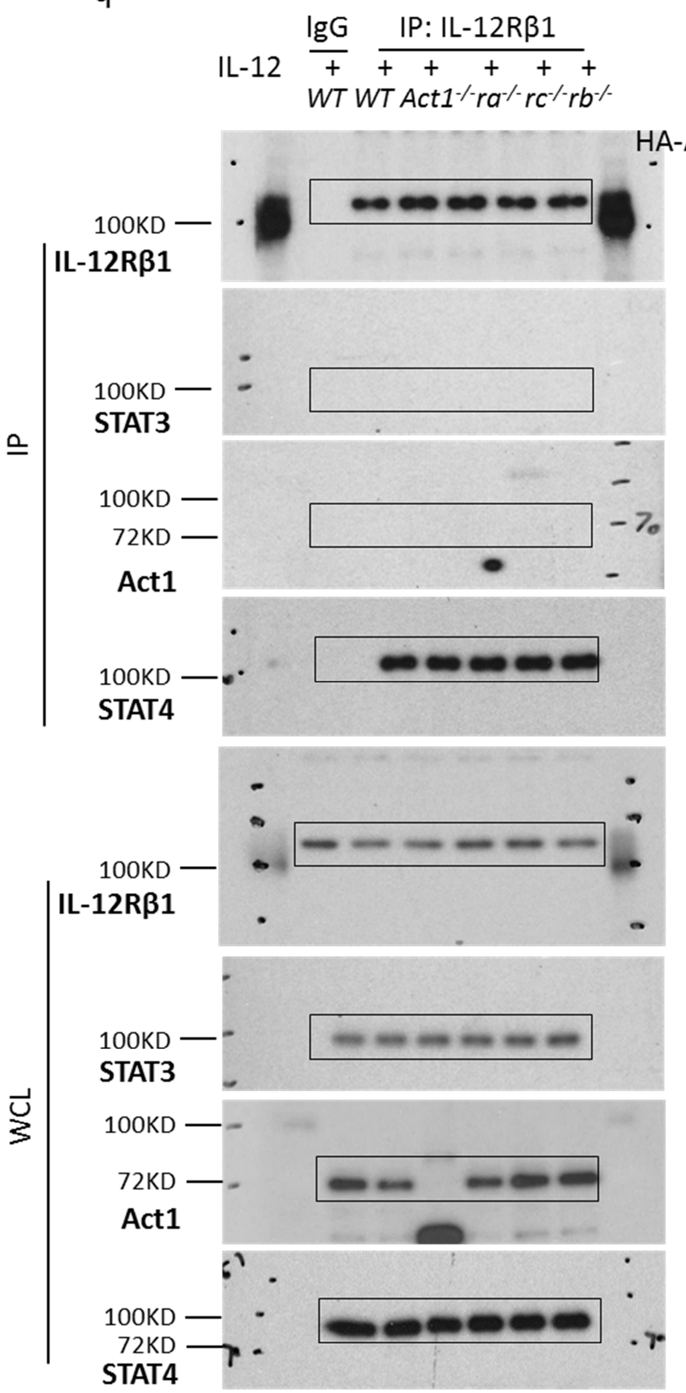

r

Supplementary Figure 2a

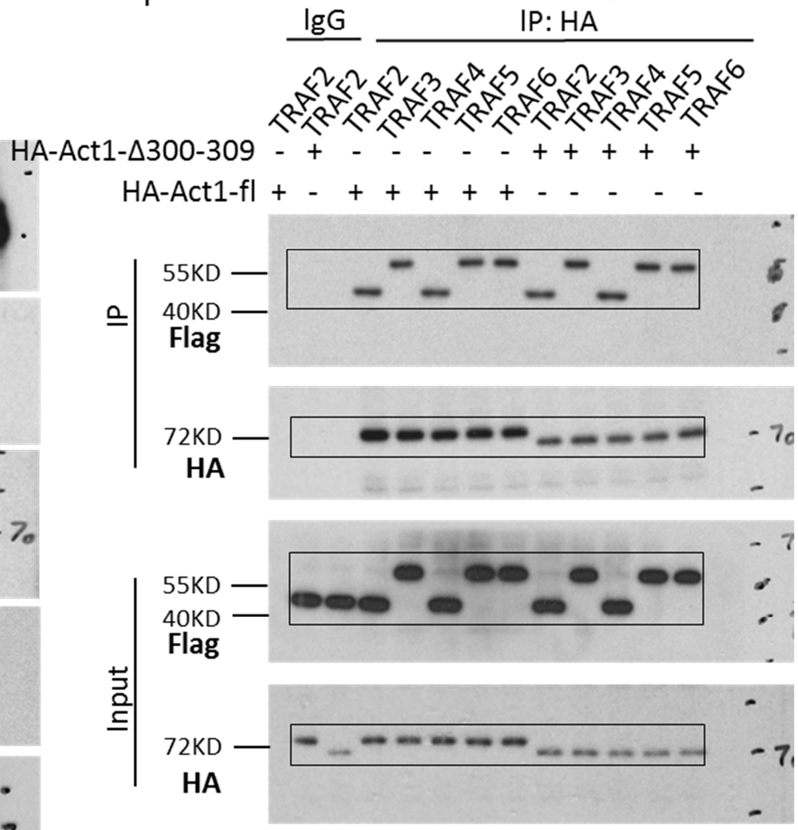

s

Supplementary Figure 2b

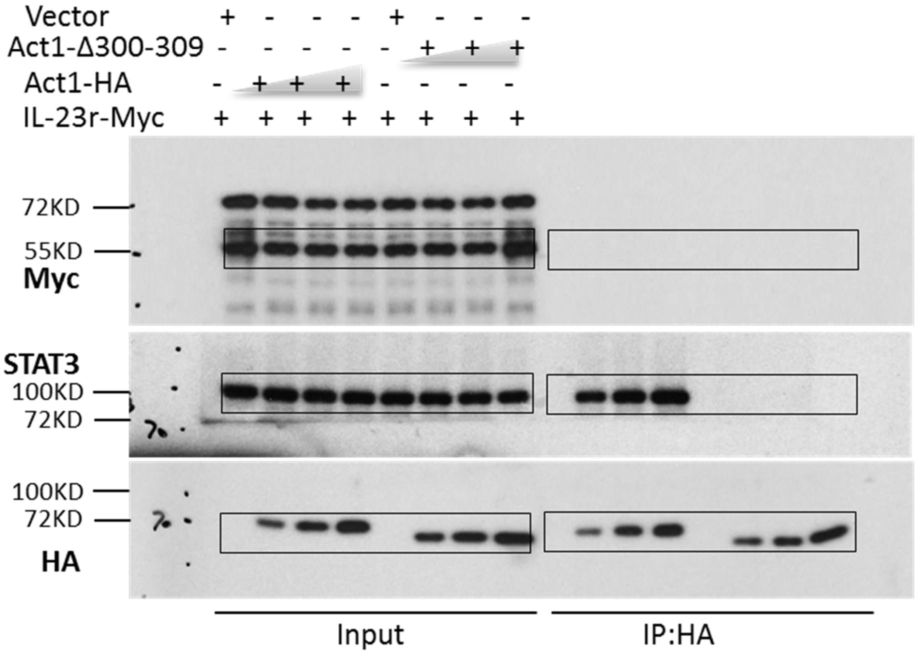

t

Supplementary Figure 3a

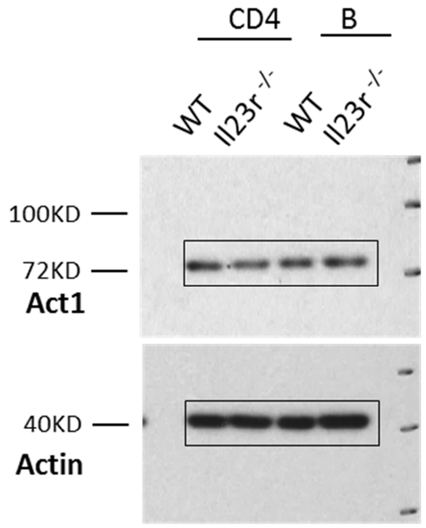

u

Supplementary Figure 2c

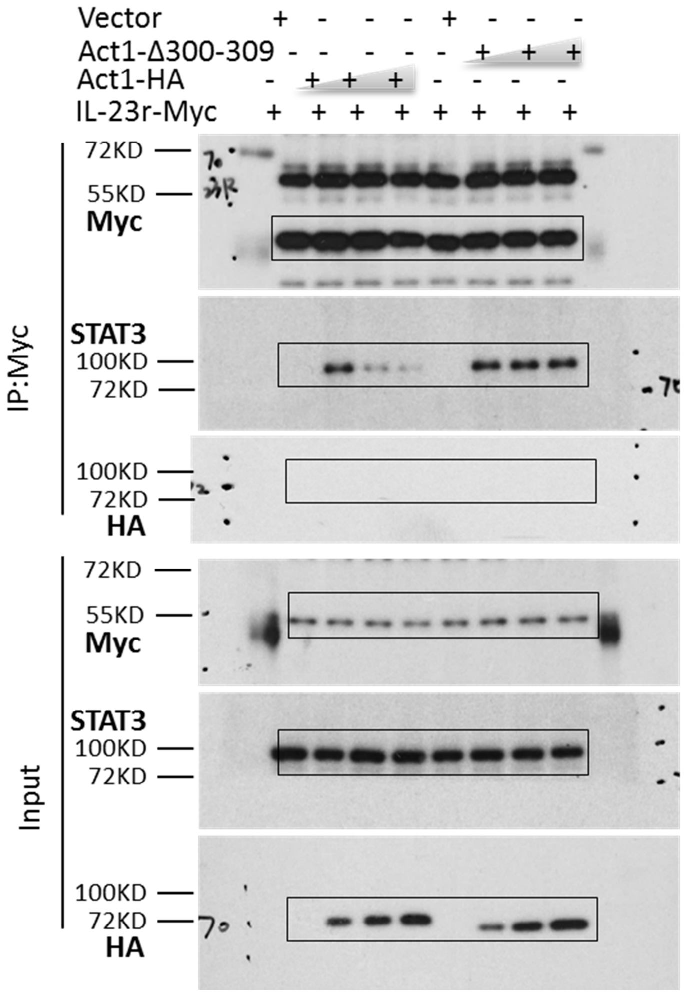

v

Supplementary Figure 4d

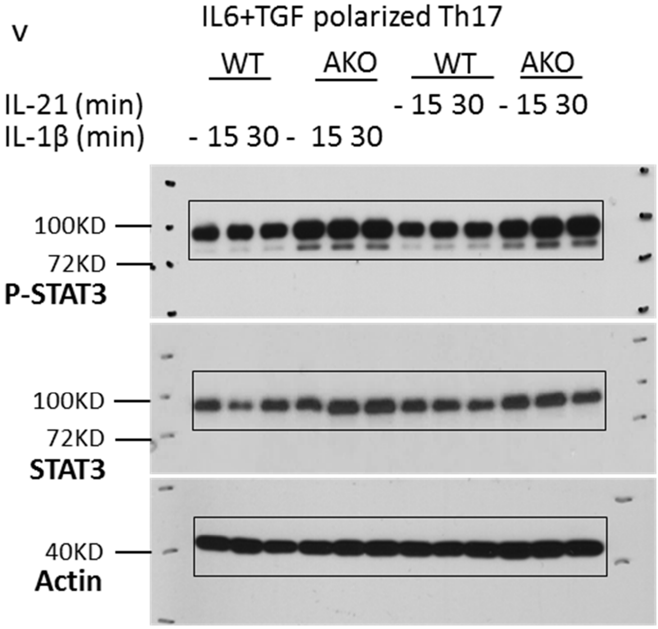

w

Supplementary Figure 4g

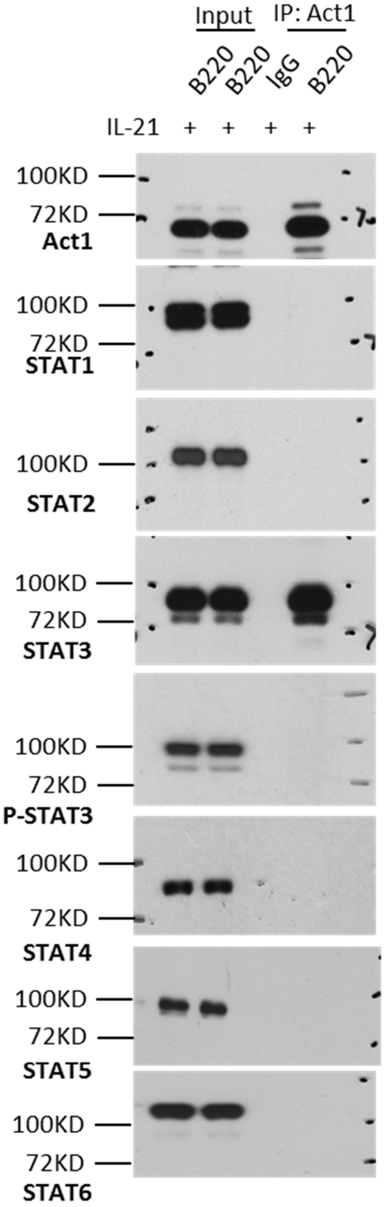

Supplementary Figure 7. Uncropped western films. (**a**) Relates to Fig. 1d. (**b**) Relates to Fig. 1e. (**c**) Relates to Fig. 2a. (**d**) Relates to Fig. 2c. (**e**) Relates to Fig. 2e. (**f**) Relates to Fig. 2g. (**g**) Relates to Fig. 2i. (**h**) Relates to Fig. 6d. (**i**) Relates to Fig. 6e. (**j**) Relates to the left panel of Fig. 7g. (**k**) Relates to the right panel of Fig. 7h. (**l**) Relates to Fig. 7h. (**m**) Relates to Supplementary Fig. 1d. (**n**) Relates to supplementary Fig. 1f. (**o**) Relates to Supplementary Fig. 1g. (**p**) Relates to Supplementary Fig. 1h. (**q**) Relates to Supplementary Fig. 1j. (**r**) Relates to Supplementary Fig. 2a. (**s**) Relates to Supplementary Fig. 2b. (**t**) Relates to Supplementary Fig. 3a. (**u**) Relates to Supplementary Fig. 2c. (**v**) Relates to Supplementary Fig. 4d. (**w**) Relates to Supplementary Fig. 4g.

REFERENCES

1. Qian Y, *et al.* Act1, a negative regulator in CD40- and BAFF-mediated B cell survival. *Immunity* **21**, 575-587 (2004).
